# Inactivation of cofilin-1 in Mcpt5-Cre-nf-Cfl1^fl/fl^ mice prevents the formation of connective tissue mast cells without affecting basophils: a new tool to investigate the specific role of CTMCs in disease

**DOI:** 10.3389/fimmu.2025.1671735

**Published:** 2026-01-28

**Authors:** Johanna Kramer, Jakob Schneider, Huiying Liu, Cinthia Silva-Vilches, Sonja Moos, Nadine Kamenjarin, Katrin Hodapp, Volodymyr Tsvilovskyy, Marc Freichel, Hans Christian Probst, Karsten Mahnke, Florian C. Kurschus, Yvonne Samstag

**Affiliations:** 1 Section Molecular Immunology, Institute of Immunology, Heidelberg University Hospital, Heidelberg, Germany; 2 Department of Dermatology, Heidelberg University Hospital, Heidelberg, Germany; 3 Institute for Immunology, University Medical Center Mainz, Mainz, Germany; 4 Research Center for Immunotherapy Forschungszentrum für Immuntherapie (FZI), University Medical Center Mainz, Mainz, Germany; 5 Institute of Pharmacology, Heidelberg University, Heidelberg, Germany

**Keywords:** actin cytoskeleton dynamics, anaphylaxis, basophils, Cofilin-1, connective tissue mast cells (CTMCs), mast cell-deficient mouse model, Mcpt5-Cre, skin inflammation

## Abstract

Actin-binding proteins play a critical role in regulating the dynamic rearrangement of the actin cytoskeleton, which is essential for maintaining cellular homeostasis and facilitating various processes in eukaryotic cells. Cofilin-1 (Cfl1), an actin-binding protein, promotes the severing and depolymerization of actin filaments. To investigate the function of Cfl1 in mast cells, we generated Mcpt5-Cre-nf-Cfl1^fl/fl^ knock-in mice, expressing a non-functional form of Cfl1 (nf-Cfl1) instead of wildtype Cfl1 under the control of the connective tissue mast cell (CTMC)-specific promoter mast cell protease 5 (Mcpt5). Expression of nf-Cfl1 resulted in the complete absence of CTMCs. Notably, normal numbers of basophils were observed, in contrast to other mast cell-deficient mice. Interestingly, an inducible knock-in of nf-Cfl1 in mature mast cells did not affect the survival of mature mast cells. The Mcpt5-Cre-nf-Cfl1^fl/fl^ mice lacking CTMCs showed impaired induction of systemic anaphylaxis. However, they remained fully susceptible to 1-fluoro-2,4-dinitrobenzene-induced contact hypersensitivity and imiquimod-induced psoriasis-like dermatitis. In addition, clearance of vaccinia virus skin infection was unaltered. Thus, this study demonstrates that CTMCs are not essential in these inflammatory skin diseases. Deviating results in some other mast cell-deficient models suggest that the concomitant lack of basophils or residual CTMCs in these mouse models influence disease outcome. Taken together, the complete absence of CTMCs and the preserved presence of basophils in Mcpt5-Cre-nf-Cfl1^fl/fl^ mice establishes this model as a valuable tool for studying the specific role of CTMCs in different diseases.

## 1 Introduction

Mast cells are strategically positioned at interfaces between the host and the environment, including the skin, gastrointestinal tract, and respiratory epithelium. Originating from hematopoietic precursors, they migrate through the bloodstream to peripheral tissues, where they complete their maturation. In mice, two distinct mast cell populations are recognized based on anatomical location and granule content: connective tissue mast cells (CTMCs) and mucosal mast cells (MMCs). CTMCs are mainly present in the peritoneal cavity and in the skin. Within the skin, CTMCs are predominantly located in the dermis, in close proximity to blood vessels and nerves.

Mast cells are best known for their role in immediate-type allergies, which can result in anaphylactic shock and can be fatal. Moreover, mast cells are involved in immune responses against parasites (1), can sense pathogens (reviewed in (2)), and have been demonstrated to be critical for antigen-specific avoidance behavior in mice (3). During type 2 responses, antigen-specific immunoglobulin E is produced, which binds to the high-affinity receptor FcϵRI expressed on the mast cell surface. Re-exposure to the antigen can induce mast cell activation and the release of their cytoplasmic granules, which are densely packed with a variety of mediators, including histamine, lysosomal enzymes, proteases, cytokines, and growth factors. The rapid release of mediators has led to the classification of mast cells as initiators of further immune response pathways.

FcϵRI-induced mast cell activation is associated with changes in cell morphology, migration, and exocytosis, which are dependent on the dynamic reorganization of the cytoskeleton (reviewed in (4)). Actin remodeling, mediated by various actin-binding proteins, is critical for many processes in mast cells. For instance, cortical actin depolymerization has been demonstrated to be important for the release of mast cell granules (5). Experiments with the RBL-2H3 histamine-releasing basophilic leukemia cell line have indicated that the actin-severing protein cofilin-1 (Cfl1) mediates actin depolymerization associated with FcϵRI-induced mast cell activation (6).

Cfl1 is a 19-kDa protein and a member of the actin depolymerizing factor/cofilin family, ubiquitously expressed in mammalian cells. Cfl1 can sever filamentous actin, thereby promoting actin depolymerization and consequently enhancing actin dynamics (7). The indispensable role of Cfl1 was demonstrated by Gurniak et al., who showed that a global knock-out of Cfl1 in mice leads to an arrest during embryonic development (8). Furthermore, T cell-specific expression of a non-functional form of Cfl1 (nf-Cfl1) in mice revealed that Cfl1 is critical for the development of αβ T cells (9).

The role of Cfl1 in mast cells remained largely unknown. Therefore, we have generated a mouse line expressing nf-Cfl1 instead of the wildtype protein specifically in CTMCs. This resulted in a CTMC-deficient mouse model without gross alterations outside the mast cell compartment. Using this mouse, we were able to show that CTMCs do not play a role in disease models of contact hypersensitivity, psoriasis, and clearance of vaccinia virus-induced skin infection.

## 2 Materials and methods

### 2.1 Mice

All animal procedures were conducted in accordance with the relevant guidelines and regulations for animal welfare set forth by the federal states of Baden-Wuerttemberg and Rhineland-Palatinate, Germany. Experiments were done with approval from the Regierungspräsidium Karlsruhe (individual animal experimentation application no. G-191/18 and no. G-234/15) or from the Landesuntersuchungsamt Rheinland-Pfalz (individual animal experimentation application no. G21-1-37).

Mice were bred and maintained at the central animal facility of the University of Heidelberg under specific pathogen-free conditions. They were provided with drinking water and food ad libitum. Mice were killed by a lethal dose of CO_2_ or cervical dislocation.

The Cre/loxP system was utilized to generate knock-in mice expressing a non-functional form of Cfl1 (B6.JM8A1.N3-Cfl1^tm1(eGFP-2A-Cfl1)Uhg^ mice; in the following termed nf-Cfl1^fl/fl^ mice) cell type-specifically. Details regarding the generation of floxed nf-Cfl1^fl/fl^ mice have been published previously (9).

In order to achieve the expression of nf-Cfl1 in CTMCs, nf-Cfl1^fl/fl^ mice were crossed with mice that expressed Cre recombinase under the control of the CTMC-specific promoter mast cell protease (Mcpt) 5 (Tg(Cma1-cre)ARoer mice; herein termed Mcpt5-Cre mice) (10). Importantly, the Mcpt5-Cre system has been extensively validated to specifically target CTMCs while sparing MMCs (10–12).

For vaccinia virus infection experiments, TCR327 mice (B6;D2-Tg(TcrLCMV)327Sdz/J, JAX stock #004694 (13)) that express the P14 transgenic T cell receptor, which recognizes the lymphocytic choriomeningitis virus glycoprotein-derived epitope GP_33–41_ in the context of H-2D^b^, were bred to B6 Thy1.1 congenic mice (B6.PL-Thy1^a^/CyJ, JAX stock #000406 (14)). The congenic marker CD90.1 allowed to identify adoptively transferred cells from TCR327 x Thy1.1 mice in host mice.

Floxed nf-Cfl1^fl/fl^ mice were mated with B6.129-Gt(ROSA)26Sor^tm1(cre/ERT2)Tyj^/J (ROSA26-CreERT2) mice (JAX stock #008463) to generate ROSA26-CreERT2-nf-Cfl1^fl/fl^ knock-in mice. In ROSA26-CreERT2-nf-Cfl1^fl/fl^ knock-in mice, the exogenous ligand 4-hydroxy tamoxifen (4-OHT) can activate the Cre recombinase, thereby enabling the inducible expression of nf-Cfl1.

### 2.2 Tissue preparation for toluidine blue staining and histological microscopy

Mouse ears and shaved dorsal skin were fixed in a 4% paraformaldehyde solution. The tissue was then dehydrated in a rising alcohol series, cleared in xylene, and embedded in paraffin. 3–6 µm tissue sections were cut using a microtome. Afterwards, the tissue sections were placed on glass slides, incubated at 96 °C for 15 min, and subsequently deparaffinized. Sections were then stained in toluidine blue staining solution (1%; made from toluidine blue O salt (Serva)) for 2 min and thoroughly rinsed with water for 5 min. The stained sections were dehydrated, embedded in Consul-Mount™, and allowed to dry for a minimum of 24 hours. Following this, the tissue sections were analyzed using a light microscope.

### 2.3 Isolation of peritoneal cells, splenocytes, lymph node cells, and basophils from blood and bone marrow

Peritoneal lavage cells were collected by flushing the peritoneal cavity of mice with phosphate buffered saline (PBS) (Sigma-Aldrich) using a plastic Pasteur pipette.

Spleens and lymph nodes were isolated and kept in ice-cold PBS until the organs were meshed through a 40 µm or 70 µm cell strainer. Red blood cells in splenocyte preparations were lysed by resuspending the cells in 2 ml of ammonium-chloride-potassium lysis buffer (Gibco) for 2 min.

Cell numbers were determined using a hemocytometer, after which cells were stained for flow cytometric analysis.

Basophils in peripheral blood and bone marrow were quantified by flow cytometry using BD Trucount^®^ tubes. Peripheral blood was collected by cardiac puncture and anticoagulated with heparin. Bone marrow was harvested from the femur and tibia of both hind limbs.

Cells were stained directly in BD Trucount^®^ tubes, followed by red blood cell lysis and flow cytometric analysis. Absolute basophil numbers were determined according to the manufacturer’s protocol.

### 2.4 Induction of nf-Cfl1 expression in mast cells and T cells *in vitro*

Peritoneal cells isolated as described in section 2.3 from homozygous ROSA26-CreERT2-nf-Cfl1^fl/fl^ knock-in mice, heterozygous ROSA26-CreERT2-nf-Cfl1^wt/fl^ knock-in mice, and nf-Cfl1^fl/fl^ control mice were cultured in the presence of interleukin (IL)-3 and SCF for a minimum of 14 days to obtain pure mast cell populations. In brief, 1 × 10^6^ peritoneal cells were cultured in a 6-well plate in 1.5 ml RPMI medium containing 20% FBS, 2 mM glutamine, 1% penicillin/streptomycin, 10 mM HEPES, and 50 µM β-mercaptoethanol in the presence of IL-3 (30 ng/ml) and SCF (20 ng/ml). Medium was refreshed when cells attained a high density, approximately after 2–3 days. After having obtained pure mast cell cultures, as confirmed by flow cytometry, 2 × 10^5^ cells were cultured in a 96-well plate in the presence of IL-3 and SCF without or with 100 nM 4-OHT.

As controls, splenic T cells were isolated from homozygous ROSA26-CreERT2-nf-Cfl1^fl/fl^ knock-in mice, heterozygous ROSA26-CreERT2-nf-Cfl1^wt/fl^ knock-in mice, and nf-Cfl1^fl/fl^ control mice by magnetic cell sorting using the Pan T cell isolation kit II from Miltenyi. In brief, splenocytes were isolated as described in section 2.3. Non-T cells were magnetically labeled and applied to LS columns in a magnetic field of a QuadroMACS™ magnet. Consequently, untouched, resting T cells were isolated. 2 × 10^6^ T cells were cultured in 96-well plates coated with plate-bound α-CD3 (5 µg/ml in PBS, clone 145-2C11) and α-CD28 (2 µg/ml in PBS, clone 37.51) antibodies. Cells were kept in RPMI medium containing 10% FBS, 2 mM glutamine, 1% penicillin/streptomycin, 10 mM HEPES, and 50 µM β-mercaptoethanol in the presence of 50 nM 4-OHT.

After 24 h, 48 h, or 72 h, living mast cells or T cells were discriminated by flow cytometric analysis using the eFluor780 viability dye (eBioscience).

### 2.5 Flow cytometry

Fluorescence-conjugated antibodies recognizing the following surface markers and molecules were used for flow cytometry: B220 (RA3-6B2), CD3 (145-2C11), CD4 (RM4–5 or GK1.5), CD8 (53-6.7), CD11b (M1/70), CD11c (N418), CD19 (6D5), CD19 (MB19-1), CD44 (IM7), CD45 (30-F11), CD49b (DX5), CD62L (MEL-14), CD69 (H1.2F3), CD90.1 (HIS51), CD103 (2E7), CD117 (2B8), CD127 (A7R34), FcεRI (MAR-1), KLRG1 (2F1), Ly6C (AL-21), Ly6G (1A8), MHCII (M5/114.15.2), NK1.1 (PK136), TCRβ (H57-597), TCRγδ (GL-3).

Surface marker staining was conducted in either FACS buffer 1 (PBS, 0.5% bovine serum albumin (BSA), 0.1% NaN_3_, Privigen (CSL Behring)) or FACS buffer 2 (PBS, 1% BSA, 20 mM EDTA, 0.02% NaN_3_), for 20 min at 4 °C. In the event that surface staining was conducted with FACS buffer 2, Fc receptors were blocked using anti-CD16/CD32 for 5 min at 4 °C. eFluor780 viability dye (eBioscience) or 7-AAD were used to discriminate between living and dead cells.

All antibodies were obtained from BD, BioLegend, Dianova, and eBioscience.

Flow cytometric measurements were performed on an LSRII (BD) or Canto (BD) and analysis was performed using FlowJo software.

### 2.6 Systemic anaphylaxis mouse model

Temperature transponders were implanted subcutaneously in sex- and age-matched mice at 9–11 weeks of age. Approximately one week later, the mice were injected intravenously with an anti-dinitrophenyl (DNP) immunoglobulin E antibody solution at a concentration of 30 µg/ml (100 µl per 25 g body weight). 24 h later, the systemic anaphylactic reaction was induced by intravenous injection of 2 mg/ml DNP-BSA (100 µl per 25 g body weight). Core body temperature was measured at five-minute intervals over a two-hour period.

### 2.7 DNFB-induced contact hypersensitivity (CHS) mouse model

Sex- and age-matched mice at the age of 8 weeks were shaved on the abdomen and 15 µl of a 0.25% 1-fluoro-2,4-dinitrobenzene (DNFB) solution in acetone/miglyol (4:1) was applied epicutaneously. Five days later, the mice were challenged with 20 µl of 0.25% DNFB solution in acetone-miglyol (4:1) per ear. The ear thickness was quantified prior to the challenge and at 6 h and 24 h post-challenge. Ear swelling was measured as the difference in ear thickness before and after the challenge (Δear thickness). As further controls, sensitized animals were challenged with solvent controls. Here, no increased ear swelling was recorded. 24 h after challenge, mice were killed and ear skin and ear-draining superficial cervical lymph nodes (*lymphonodus parotideus superficialis*) were isolated for flow cytometric analysis of surface markers as described in section 2.5. Lymph node cells were prepared as described in section 2.3. Ear skin was digested in PBS with 0.5% trypsin and 5 mM EDTA for 30 min at 37 °C. Digestion was stopped by adding PBS containing 20% FBS and 0.1% DNase (Roche). Thereafter, the tissue was homogenized and passed through a 70 µm cell strainer.

### 2.8 Imiquimod (IMQ)-induced psoriasis mouse model

Sex- and age-matched mice at the age of 7 weeks were shaved and depilated on the lower back. Two days later, daily treatment with Aldara cream containing 5% IMQ (Meda Pharma) or with sham cream (containing all ingredients except IMQ, Pharmacy of University Hospital Heidelberg) was started for five consecutive days. 50 mg cream was applied on the dorsal skin and 5 mg cream was applied on each ear. The thickness of the back skin, ear thickness, and body weight were measured on a daily basis using an electronic external measuring gauge. Additionally, scaling and erythema formation were scored daily from 0 to 4, whereby 0 indicates no severity and 4 indicates high severity. The cumulative score was calculated as described previously (15).

At day 6, mice were killed, and ear skin and ear-draining superficial cervical lymph nodes (*lymphonodus parotideus superficialis*) were isolated for analysis by flow cytometry. Lymph nodes were prepared as described in section 2.3. Ears were split into dorsal and ventral parts, mechanically disrupted, and digested using 0.25 mg/ml Liberase (Roche) and 0.05 mg/ml DNase (Roche). Subsequently, the cell suspension was filtered through a 40 µm cell strainer before the cells were stained for flow cytometry as described in section 2.5. For intracellular cytokine staining, cells isolated from ears were restimulated with 50 ng/ml PMA (Sigma-Aldrich) and 500 ng/ml ionomycin (Sigma-Aldrich) in the presence of 1 µg/ml Brefeldin A (Sigma-Aldrich) for 4 h at 37 °C. Subsequently, surface markers were stained as described in section 2.5. Following fixation with 2% ROTI® HistoFix (Roth) in PBS for 40 min at 4 °C, intracellular staining of IL-17A (eBio17B7) was conducted in Permeabilization Buffer (eBioscience) overnight at 4 °C (as described in (16)).

### 2.9 Vaccinia virus-induced skin infection

Recombinant vaccinia virus expressing the lymphocytic choriomeningitis virus glycoprotein-derived epitope GP_33–41_ (17) was propagated on BSC-40 cells at a low multiplicity of infection.

Sex- and age-matched mice between 6 and 21 weeks of age were administered 1 × 10^5^ splenic naïve TCR327^+^ CD8^+^ T cells in HBSS via intravenous injection. For this purpose, splenic CD8^+^ T cells were isolated by magnetic cell separation from TCR327 x Thy1.1 congenic mice.

One day later, 2 × 10^6^ plaque-forming units of vaccinia virus in 10 μl DMEM (10% FBS, 1% L-glutamine, 1% penicillin/streptomycin, 1% sodium pyruvate, 50 µM β-mercaptoethanol) were applied onto the mouse ear pinnae. Virus-coated ear pinnae were punctured 25 times with a 27G 1/2”-gauge injection needle. On the day of analysis, mice were killed and a tissue sample (3 mm × 3 mm) of infected ears was taken for virus titer analysis. The ear skin, skin-draining lymph nodes, and blood were isolated for flow cytometric analysis of immune cells and restimulation assay. To isolate cells from the ear skin, ears were split into dorsal and ventral portions and afterwards cut into small pieces. Digestion was conducted in RPMI 1640 containing 800 U/ml collagenase type IV (Worthington) and 50 U/ml DNase I (Sigma-Aldrich) for 90 min at 37 °C. Subsequently, the cell suspension was filtered through a 70 µm cell strainer. Lymph node cells were isolated as described in section 2.3. Blood was collected from the submandibular vein using a lancet and erythrocytes were removed by osmotic lysis.

Virus titers were determined by plaque assay on BSC-40 cells. Cell suspensions were titrated by tenfold dilutions onto subconfluent BSC-40 monolayers in a 24-well plate and incubated for 1 h at room temperature. Subsequently, DMEM was added and the incubation process was continued at 37 °C. After 24 h of incubation, the medium was removed and the cells were stained with crystal violet solution. The number of plaques was counted and plaque-forming units were determined.

Intracellular cytokine staining was performed on TCR327^+^ CD8^+^ T cells from the skin-draining lymph nodes of virus-infected mice. The cells were restimulated in IMDM medium (10% FBS, 1% L-glutamine, 1% penicillin/streptomycin, 1% sodium pyruvate, 50 µM β-mercaptoethanol) containing 1 µM GP_33–41_ peptide and 5 µg/ml Brefeldin A in the presence of 25 U/ml IL-2 for 6 h. Subsequently, the cells were stained for flow cytometric analysis of intracellular cytokines using antibodies against IFN-γ (XMG1.2), TNF-α (JES6-5H4), and IL-2 (MP6-XT22).

### 2.10 Statistics

Prism 10 was employed for the purpose of statistical analysis. Values are expressed as mean ± standard error of the mean (SEM). Unpaired Student’s t-test was used to determine significant differences in mean values between two groups. In the event that more than two groups were analyzed, statistical significance was determined using either one-way analysis of variance with a Bonferroni *post hoc* test or with a Kruskal-Wallis test followed by Dunn’s multiple comparison test. Probability values (p values) of p ≤ 0.05 (*), p ≤ 0.01 (**), p ≤ 0.001 (***), and p < 0.0001 (****) were considered statistically significant.

## 3 Results

### 3.1 Generation of mice expressing nf-Cfl1 specifically in mast cells

**Figure 1:**
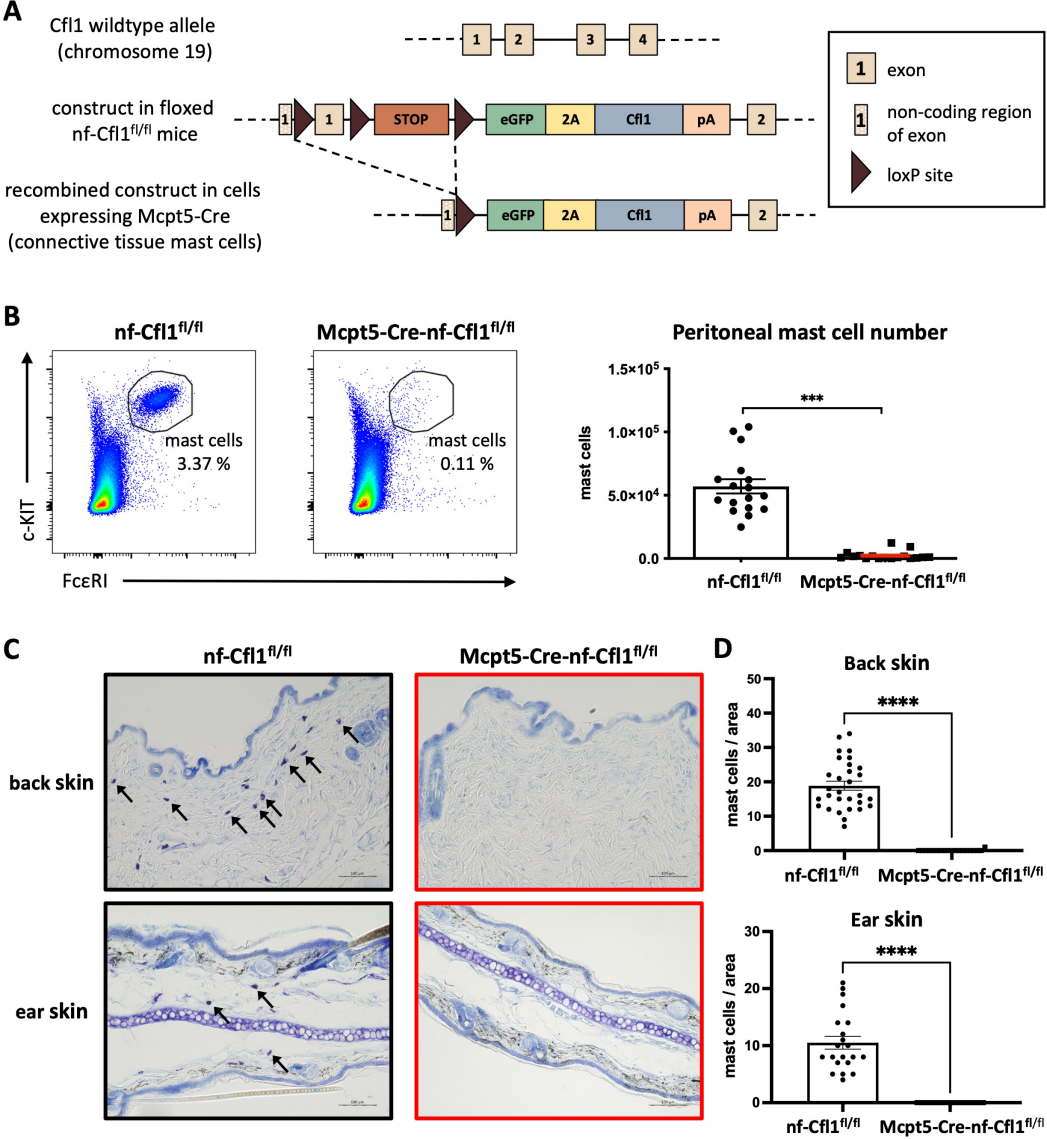
Mcpt5-Cre-nf-Cfl1^fl/fl^ mice lack CTMCs in the peritoneum and in the skin. **(A)** The wildtype mouse Cfl1 gene, located on chromosome 19, is composed of four exons (first line). Exon 1 encompasses the ATG start codon. The gene was targeted by inserting a floxed stop cassette and an eGFP-2A-Cfl1 sequence into the intronic region between exon 1 and exon 2. Furthermore, an additional loxP site was introduced into the non-coding region of exon 1 (second line). Floxed mice were crossed with Mcpt5-Cre mice to achieve mast cell-specific knock-out of endogenous Cfl1 by deleting exon 1 and simultaneous knock-in of the eGFP-2A-Cfl1 expression cassette, which allows concomitant expression of eGFP and non-functional Cfl1. **(B)** Left: Peritoneal lavage cells from nf-Cfl1^fl/fl^ control mice and Mcpt5-Cre-nf-Cfl1^fl/fl^ knock-in mice were analyzed by flow cytometry for the presence of c-KIT^+^ FcεRI^+^ mast cells. Right: Bar graph shows peritoneal lavage cell numbers of nf-Cfl1^fl/fl^ control mice (black) and of Mcpt5-Cre-nf-Cfl1^fl/fl^ knock-in mice (red). Data in the bar graph are represented as mean ± SEM and summarize results from 10 independent experiments with a total of 17 mice per group. Unpaired Student’s t-test was used to determine significant differences between groups. Differences of p ≤ 0.05 were considered to be statistically significant (*** p ≤ 0.001). **(C)** Ear and back skin sections of nf-Cfl1^fl/fl^ control mice (black) and Mcpt5-Cre-nf-Cfl1^fl/fl^ knock-in mice (red) were stained for mast cells using toluidine blue. Tissue sections measuring 3–6 µm in thickness were analyzed using a light microscope. Arrows indicate the presence of mast cells. Scale bars represent 100 µm. Representative pictures from one out of two independent experiments with in total 3 mice per group are shown. **(D)** Quantification and statistical evaluation of mast cell numbers in back skin and ear skin. Briefly, mast cells were identified by toluidine blue staining as shown in **(C)** and counted manually using ImageJ in ≥ 7 non-overlapping ear skin sections, or ≥ 8 non-overlapping back skin sections per mouse. In total 3 animals per group were analyzed. Data are presented as mean ± SEM and were compared using an unpaired t-test (****p ≤ 0.0001).

The actin-severing protein Cfl1 plays a crucial role in numerous cellular processes. Knock-out of endogenous Cfl1 and expression of nf-Cfl1 under the control of the T cell-specific Lck promoter resulted in the generation of a mouse model that was deficient in αβ T cells but not in γδ T cells (9). To decipher the significance of Cfl1 for CTMCs, floxed nf-Cfl1^fl/fl^ mice (described in detail in (9)) were crossed with Mcpt5-Cre mice, which express Cre under the control of the Mcpt5 promoter specifically active in CTMCs (10) (**Figure 1A**). In the resulting Mcpt5-Cre-nf-Cfl1^fl/fl^ mice, wildtype Cfl1 is knocked out upon Cre-mediated recombination due to disruption of exon 1 in Cre-expressing cells. At the same time, the complementary DNAs encoding enhanced green fluorescent protein (eGFP) and nf-Cfl1 are knocked in. These complementary DNAs are transcribed as one single messenger RNA by the use of a viral 2A sequence. Cfl1 expressed from the introduced complementary DNAs carries an additional proline residue at the N-terminus, derived from the viral 2A sequence. This prevents co- and post-translational processing of the protein and renders it non-functional. Consequently, instead of the wildtype protein, nf-Cfl1 is expressed in CTMCs of Mcpt5-Cre-nf-Cfl1^fl/fl^ mice.

Homozygous knock-in mice (Mcpt5-Cre-nf-Cfl1^fl/fl^) and homozygous control mice that do not express Cre (nf-Cfl1^fl/fl^ mice) were compared to each other. The genotype of the mice was analyzed by polymerase chain reaction of tissue biopsies (**Supplementary Figures 1A, B**). No obvious phenotypic abnormalities were observed in nf-Cfl1^fl/fl^ and Mcpt5-Cre-nf-Cfl1^fl/fl^ mice.

### 3.2 Expression of nf-Cfl1 under the control of the Mcpt5 promoter in Mcpt5-Cre-nf-Cfl1^fl/fl^ mice led to absence of CTMCs in the peritoneum and the skin

In order to determine the presence of CTMCs in Mcpt5-Cre-nf-Cfl1^fl/fl^ mice, the emergence of peritoneal mast cells was analyzed by flow cytometry by determining the surface markers c-KIT and FcεRI which are typically expressed on mast cells. As illustrated in the dot plots, a discrete c-KIT^+^ FcεRI^+^ mast cell population was observed in nf-Cfl1^fl/fl^ control mice, whereas this double-positive population was absent in peritoneal lavage cells from Mcpt5-Cre-nf-Cfl1^fl/fl^ knock-in mice (**Figure 1B**, left panel). The average number of peritoneal mast cells present in nf-Cfl1^fl/fl^ control mice was 57,000. In contrast, only a negligible number of peritoneal mast cells was observed in Mcpt5-Cre-nf-Cfl1^fl/fl^ mice (**Figure 1B**, right panel).

Mast cells constitute approximately 10% of leukocytes in the mouse ear skin (18). The presence of mast cells in the skin of Mcpt5-Cre-nf-Cfl1^fl/fl^ mice was examined through toluidine blue staining of back and ear skin sections. The analysis revealed a substantial presence of mast cells in the skin of nf-Cfl1^fl/fl^ control mice, whereas the skin of Mcpt5-Cre-nf-Cfl1^fl/fl^ knock-in mice displayed a complete absence of mast cells (**Figures 1C, D**).

These data show that the homozygous expression of nf-Cfl1 instead of wildtype Cfl1 in mast cells resulted in CTMC ablation.

### 3.3 Mcpt5-Cre-nf-Cfl1^fl/fl^ mice were not sensitive to the induction of systemic anaphylaxis

Following the demonstration that mast cells were phenotypically absent in the peritoneum and the skin of Mcpt5-Cre-nf-Cfl1^fl/fl^ mice, a model was required to also functionally assess the lack of mast cells. Since it is well known that CTMCs are critical effector cells in immunoglobulin E-dependent anaphylaxis (19) (reviewed in (20)), an *in vivo* model of passive systemic anaphylaxis was conducted. Thus, mice were injected intravenously with anti-DNP immunoglobulin E antibody solution and 24 h later with DNP-BSA to induce the anaphylactic reaction. The body temperature of the mice was monitored for 2 h.

**Figure 2:**
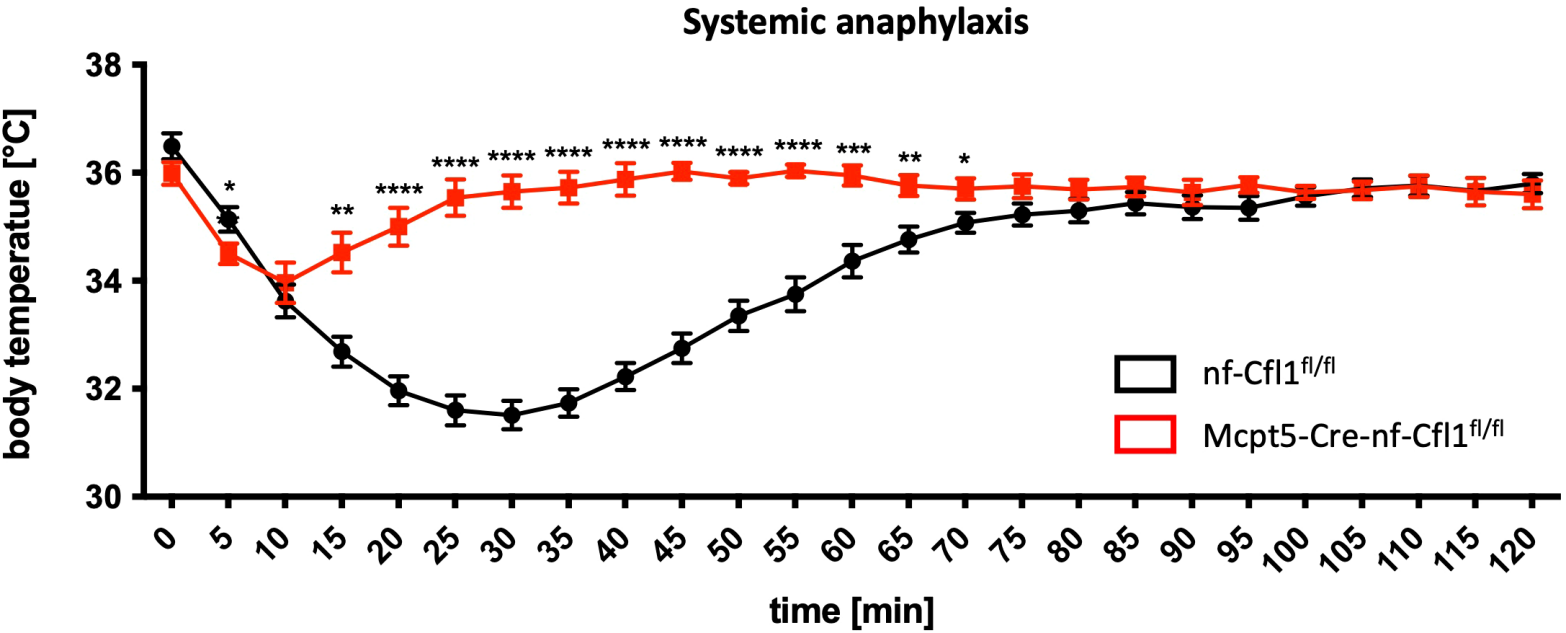
Absence of systemic anaphylactic reaction in Mcpt5-Cre-nf-Cfl1^fl/fl^ mice indicates functional absence of mast cells. Time course of the body temperature of nf-Cfl1^fl/fl^ control mice (black) and Mcpt5-Cre-nf-Cfl1^fl/fl^ knock-in mice (red) during systemic anaphylaxis elicited by injection of dinitrophenyl-bovine serum albumin at time point “0”. Mice were injected intravenously with anti-dinitrophenyl immunoglobulin E 24 h before. Data in the graph are represented as mean ± SEM and summarize results from 2 independent experiments with a total of 8 mice per group. Unpaired Student’s t-test was used to determine significant differences between groups. Differences of p ≤ 0.05 were considered to be statistically significant (*p ≤ 0.05; **p ≤ 0.01; ***p ≤ 0.001; ****p < 0.0001).

Nf-Cfl1^fl/fl^ control mice exhibited a temperature curve typical for systemic anaphylaxis (**Figure 2**): Immediately following the administration of DNP-BSA, which induced the systemic anaphylactic reaction, the body temperature began to decline from its initial value of 36.5 °C, reaching a minimum of 31.5 °C 30 min after DNP-BSA administration. Subsequently, the temperature increased again but did not reach the original value within the measurement timeframe. The temperature curve of mast cell-deficient Mcpt5-Cre-nf-Cfl1^fl/fl^ mice exhibited a markedly disparate profile. The initial body temperature was 36 °C and declined to a minimum of 34 °C within the first 10 min following DNP-BSA administration. Afterwards, the temperature increased again rapidly. It is noteworthy that the initial temperature decline observed in Mcpt5-Cre-nf-Cfl1^fl/fl^ mice is a characteristic response to the anesthesia administered prior to DNP application, as consistently reported in previous literature (21, 22). At each measurement point between 15 min and 70 min following DNP application, the body temperature of Mcpt5-Cre-nf-Cfl1^fl/fl^ mice was significantly higher than that of nf-Cfl1^fl/fl^ mice. Hence, the lack of an anaphylactic reaction confirms CTMC absence in Mcpt5-Cre-nf-Cfl1^fl/fl^ mice also functionally.

### 3.4 Other immune cell populations, including basophils, were not affected in Mcpt5-Cre-nf-Cfl1^fl/fl^ mice

Next, immune cell populations in lymph nodes and the spleen of Mcpt5-Cre-nf-Cfl1^fl/fl^ and nf-Cfl1^fl/fl^ mice were analyzed.

**Figure 3:**
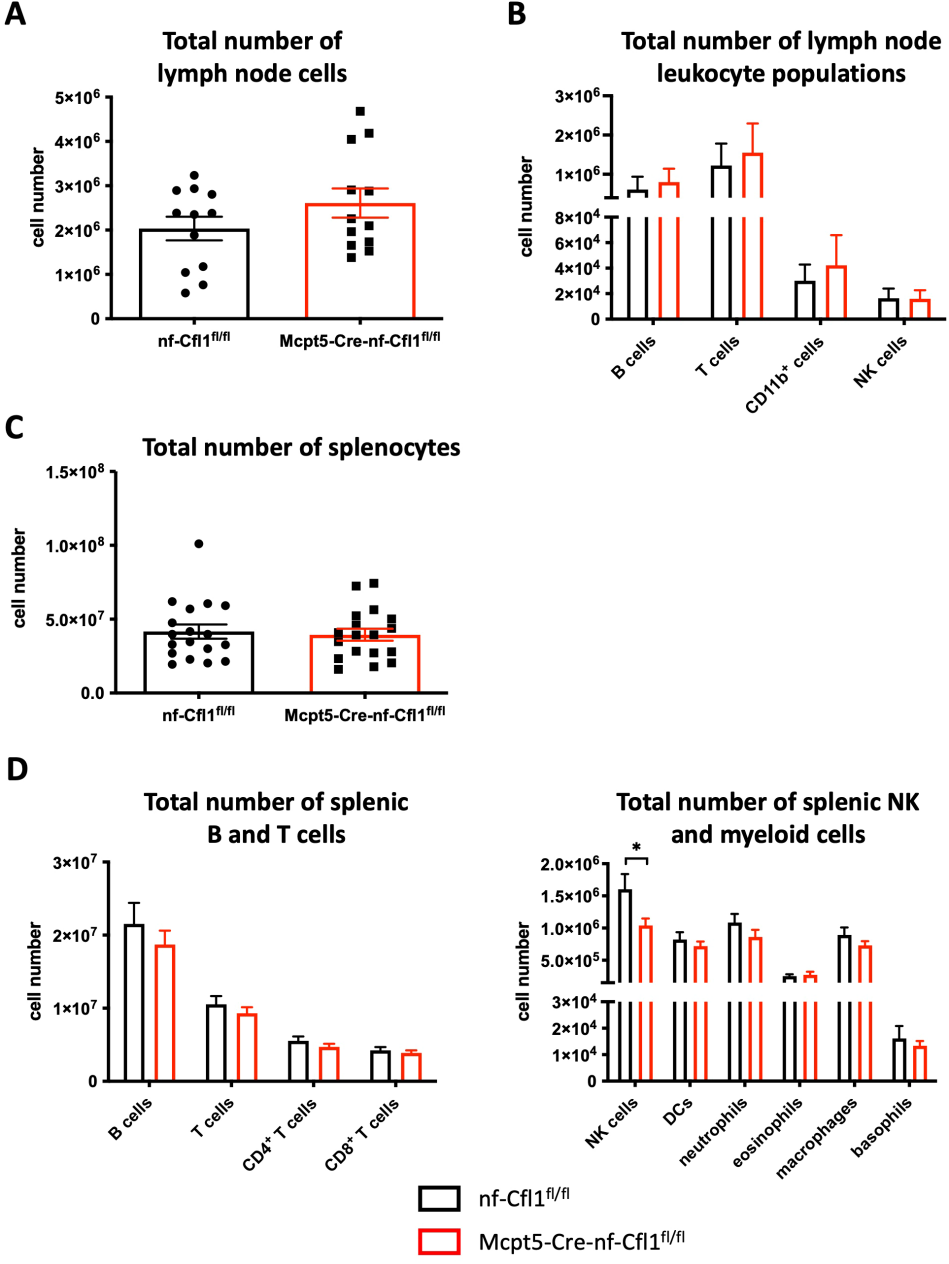
Except splenic NK cells, Mcpt5-Cre-nf-Cfl1^fl/fl^ mice have normal numbers of other immune cells including basophils in the spleen and lymph nodes. **(A)** Total cell number of inguinal and axillary lymph nodes in nf-Cfl1^fl/fl^ control mice (black) and Mcpt5-Cre-nf-Cfl1^fl/fl^ knock-in mice (red). Data summarize 7 independent experiments with a total of 12 mice per group. **(B)** Total numbers of lymph node immune cell populations from nf-Cfl1^fl/fl^ control mice (black) and Mcpt5-Cre-nf-Cfl1^fl/fl^ knock-in mice (red) were analyzed by flow cytometry. Data summarize 7 independent experiments with a total of 12 mice per group. **(C)** Total splenocyte numbers in nf-Cfl1^fl/fl^ control mice (black) and Mcpt5-Cre-nf-Cfl1^fl/fl^ knock-in mice (red). Data summarize 10 independent experiments with a total of 18 mice per group. **(D)** Total numbers of splenic immune cell populations from nf-Cfl1^fl/fl^ control mice (black) and Mcpt5-Cre-nf-Cfl1^fl/fl^ knock-in mice (red) were analyzed by flow cytometry. Data summarize ≥ 4 independent experiments with a total of ≥ 7 mice per group. Data in the bar graphs are represented as mean ± SEM. Unpaired Student’s t-test was used to determine significant differences between groups. Differences of p ≤ 0.05 were considered to be statistically significant (*p ≤ 0.05).

The total cell numbers in inguinal and axillary lymph nodes were comparable between nf-Cfl1^fl/fl^ and Mcpt5-Cre-nf-Cfl1^fl/fl^ mice (**Figure 3A**). Likewise, the absolute numbers of B cells, T cells, CD11b^+^ cells, and NK cells in the lymph nodes were unaltered (**Figure 3B**).

The total splenocyte number was also comparable between nf-Cfl1^fl/fl^ and Mcpt5-Cre-nf-Cfl1^fl/fl^ mice (**Figure 3C**). A similar observation was made for the total numbers of B cells, T cells (including CD4^+^ and CD8^+^ T cells), dendritic cells (DCs), neutrophils, eosinophils, and macrophages (**Figure 3D**). Yet, in contrast to the lymph nodes, the total number of NK cells in the spleen was slightly diminished in Mcpt5-Cre-nf-Cfl1^fl/fl^ mice (1.04 × 10^6^) compared to nf-Cfl1^fl/fl^ control mice (1.6 × 10^6^).

Importantly, the total number of splenic basophils was not affected by the absence of CTMCs in Mcpt5-Cre-nf-Cfl1^fl/fl^ mice (**Figure 3D**). Consistently, no significant differences in basophil numbers were detected in the blood and bone marrow of Mcpt5-Cre-nf-Cfl1^fl/fl^ mice compared to control animals (**Supplementary Figure 2**).

Consequently, with the exception of slightly diminished numbers of NK cells in the spleen, expression of nf-Cfl1 under the control of the Mcpt5 promoter resulted in the generation of a mouse model selectively lacking CTMCs.

### 3.5 Development but not survival of mast cells is dependent on Cfl1 function

**Figure 4:**
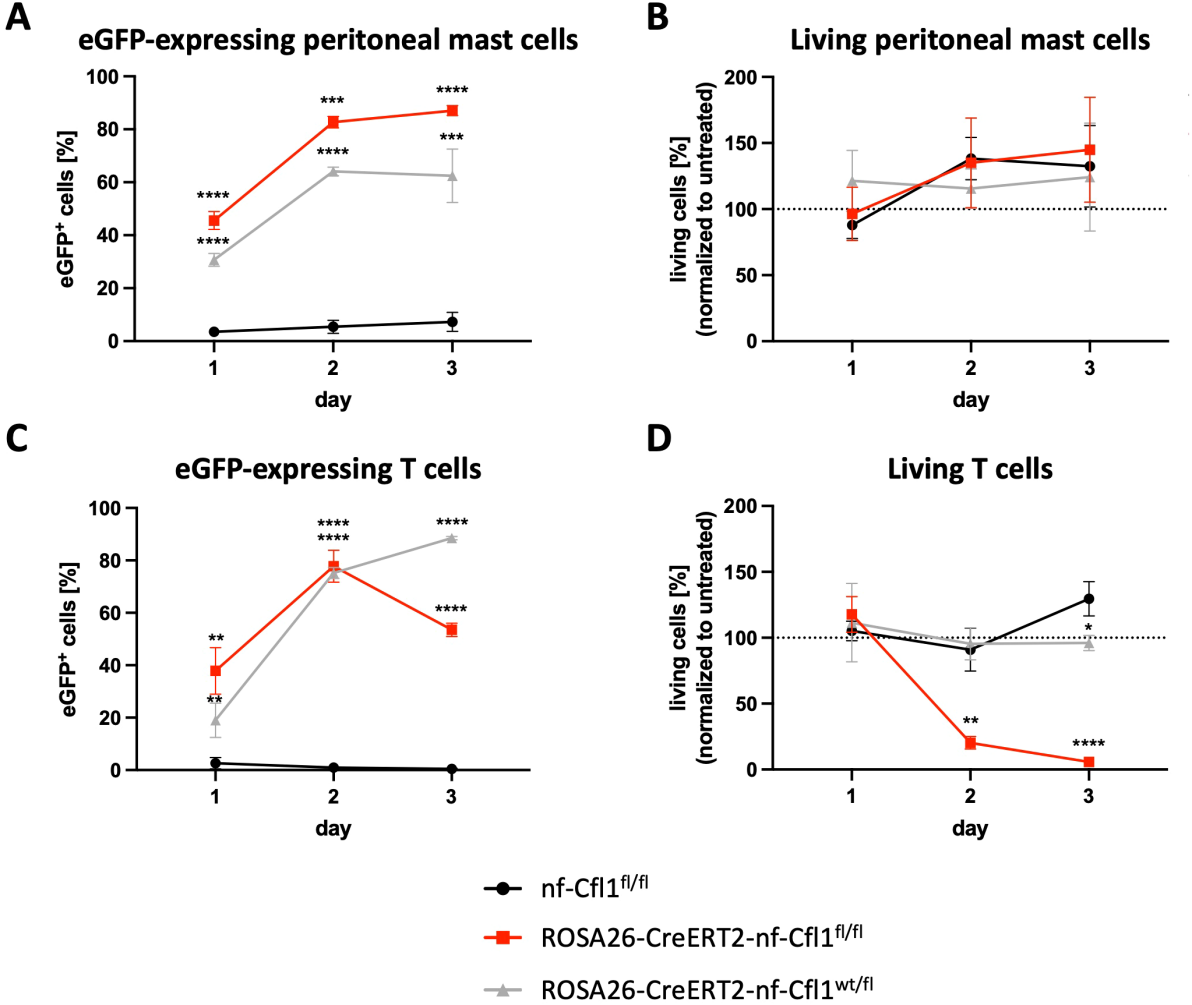
Functional Cfl1 is essential for the survival of mature T cells but not for the viability of mature CTMC-like peritoneal mast cells. Peritoneal cells and splenic T cells were isolated from homozygous ROSA26-CreERT2-nf-Cfl1^fl/fl^ knock-in mice, heterozygous ROSA26-CreERT2-nf-Cfl1^wt/fl^ knock-in mice, and nf-Cfl1^fl/fl^ control mice. Mature CTMC-like peritoneal mast cells and splenic T cells were cultured with 100 nM or 50 nM 4-hydroxy tamoxifen, respectively, for a period of three days. **(A)** eGFP expression in peritoneal mast cells was analyzed by flow cytometry. **(B)** The amount of living mast cells was determined by flow cytometric analysis using eFluor780 (eBioscience). **(C)** eGFP expression in T cells was analyzed by flow cytometry. **(D)** The amount of living T cells was determined by flow cytometric analysis using eFluor780 (eBioscience). Data in the graphs are represented as mean ± SEM and summarize results from 4 independent experiments with a total of ≥ 4 mice per group. One-way analysis of variance with a Bonferroni post hoc test was used to determine significant differences between ROSA26-CreERT2-nf-Cfl1^fl/fl^ mice or ROSA26-CreERT2-nf-Cfl1^wt/fl^ mice and nf-Cfl1^fl/fl^ control mice. Differences of p ≤ 0.05 were considered to be statistically significant (*p ≤ 0.05; **p ≤ 0.01; ***p ≤ 0.001; ****p < 0.0001).

The absence of adult CTMCs in Mcpt5-Cre-nf-Cfl1^fl/fl^ mice led to the question whether the development of CTMCs was abrogated or if adult CTMCs underwent cell death. To address this question, ROSA26-CreERT2 mice were utilized, which enable the temporal regulation of Cre expression via tamoxifen administration. Peritoneal cells isolated from ROSA26-CreERT2-nf-Cfl1^fl/fl^ knock-in mice, ROSA26-CreERT2-nf-Cfl1^wt/fl^ knock-in mice, and nf-Cfl1^fl/fl^ control mice were cultured in the presence of IL-3 and SCF for a minimum of 14 days to obtain pure mast cell populations. Expression of nf-Cfl1 was induced in peritoneal mast cells *in vitro* by treatment with 4-OHT for three days. The induction of nf-Cfl1 expression from the inserted construct was confirmed by the presence of eGFP over time. Following 4-OHT treatment for two days, approximately 80% of peritoneal mast cells isolated from homozygous ROSA26-CreERT2-nf-Cfl1^fl/fl^ knock-in mice expressed eGFP, an amount that increased further after three days (**Figure 4A**). Approximately 60% of peritoneal mast cells isolated from heterozygous ROSA26-CreERT2-nf-Cfl1^wt/fl^ knock-in mice expressed eGFP, while eGFP expression in peritoneal mast cells from nf-Cfl1^fl/fl^ control mice was negligible. Inducing either homozygous expression of nf-Cfl1 in peritoneal mast cells isolated from ROSA26-CreERT2-nf-Cfl1^fl/fl^ knock-in mice or heterozygous expression of nf-Cfl1 in peritoneal mast cells isolated from ROSA26-CreERT2-nf-Cfl1^wt/fl^ knock-in mice did not affect mast cell viability. It remained similar to the viability of peritoneal mast cells cultured from nf-Cfl1^fl/fl^ control mice (**Figure 4B**).

The functionality of the ROSA26-CreERT2-nf-Cfl1^fl/fl^ knock-in mice was proven by culturing splenic T cells in the presence of 4-OHT. Earlier we had shown that T cell-specific expression of nf-Cfl1 in knock-in mice led to the absence of αβ T cells (9). To determine whether expression of nf-Cfl1 affects the viability of mature T cells, experiments were performed with T cells purified from the above mentioned ROSA26-CreERT2 mice. After two days of treatment with 4-OHT, over 70% of the T cells isolated from ROSA26-CreERT2-nf-Cfl1^fl/fl^ and ROSA26-CreERT2-nf-Cfl1^wt/fl^ knock-in mice expressed eGFP, indicating the expression of nf-Cfl1 from the inserted construct (**Figure 4C**). The induction of homozygous expression of nf-Cfl1 *in vitro* led to a drastically decreased portion of T cells after two days: Approximately 80% of the T cells were found to be non-viable after two days. By the end of the three-day observation period, nearly the entire T cells were dead. In contrast, heterozygous expression of nf-Cfl1 in T cells from ROSA26-CreERT2-nf-Cfl1^wt/fl^ knock-in mice did not result in increased T cell death (**Figure 4D**).

Taken together, these results demonstrate that functional Cfl1 is essential for the survival of mature T cells but not for the viability of CTMCs. The lack of CTMCs in Mcpt5-Cre-nf-Cfl1^fl/fl^ mice is, therefore, a consequence of impaired mast cell development due to the absence of functional Cfl1.

### 3.6 Mcpt5-Cre-nf-Cfl1^fl/fl^ mice showed a normal reaction in an *in vivo* model of DNFB-induced contact hypersensitivity in the skin

**Figure 5:**
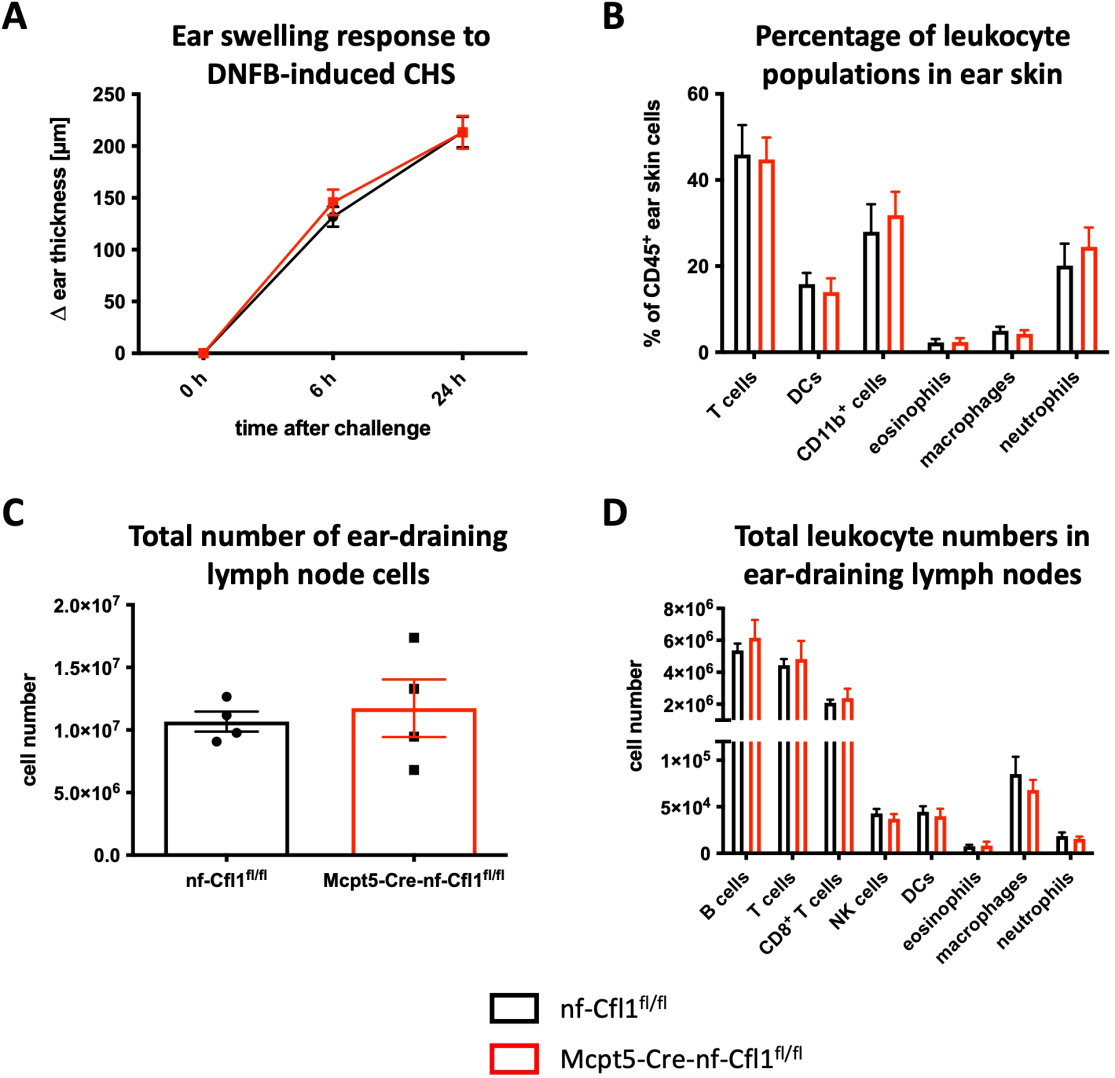
Normal reaction of Mcpt5-Cre-nf-Cfl1^fl/fl^ mice to DNFB-induced CHS. Mice were sensitized with 0.25% DNFB 5 days prior to challenge with 0.25% DNFB. **(A)** Ear swelling of nf-Cfl1^fl/fl^ control mice (black) and Mcpt5-Cre-nf-Cfl1^fl/fl^ knock-in mice (red) was measured at 6 h and 24 h following challenge with DNFB. **(B–D)**: Mice were killed 24 h after DNFB challenge and ear skin **(B)** as well as ear-draining lymph nodes (**C** + **D**) were analyzed by flow cytometry to determine the presence of immune cells. Data in the graphs are represented as mean ± SEM and summarize results from 2 independent experiments with a total of 7 mice per group (**A** + **B**) or 1 experiment with a total of 4 mice per group (**C** + **D**). Unpaired Student’s t-test was used to determine significant differences between groups. Differences of p ≤ 0.05 were considered to be statistically significant.

To address the role of mast cells in CHS, Mcpt5-Cre-nf-Cfl1^fl/fl^ mice were subjected to DNFB-induced CHS, a model for delayed-type hypersensitivity response. The ear thickness of both Mcpt5-Cre-nf-Cfl1^fl/fl^ mice and nf-Cfl1^fl/fl^ mice exhibited an equivalent increase of approximately 145 µm 6 h after challenge (**Figure 5A**). 24 h after challenge, the ear thickness had increased further, reaching approximately 215 µm. However, no significant differences in the ear thickness were observed between Mcpt5-Cre-nf-Cfl1^fl/fl^ mice and nf-Cfl1^fl/fl^ mice. 24 h after challenge, immune cell populations in ear skin and ear-draining lymph nodes were analyzed by flow cytometry. The frequency of T cells, DCs, CD11b^+^ cells, eosinophils, macrophages, and neutrophils in the ear skin was similar in Mcpt5-Cre-nf-Cfl1^fl/fl^ mice and nf-Cfl1^fl/fl^ mice (**Figure 5B**). The total cell number in ear-draining lymph nodes and the total number of all analyzed immune cell populations in ear-draining lymph nodes (B cells, T cells, NK cells, DCs, eosinophils, macrophages, and neutrophils) were also comparable between Mcpt5-Cre-nf-Cfl1^fl/fl^ and nf-Cfl1^fl/fl^ mice (**Figures 5C, D**). Therefore, CTMCs do not appear to be involved in DNFB-induced CHS in the present mouse model of mast cell deficiency.

### 3.7 Development of IMQ-induced psoriasis is not influenced by the absence of CTMCs in Mcpt5-Cre-nf-Cfl1^fl/fl^ mice

The cytokine IL-17 plays a central role in the pathogenesis of psoriasis, a chronic inflammatory skin disease. In addition to Th17 cells and IL-17-producing γδ T cells, mast cells have been demonstrated to function as IL-17-producers in both normal and psoriatic human skin (23). Nevertheless, the precise role of mast cells in psoriasis remains unclear.

**Figure 6:**
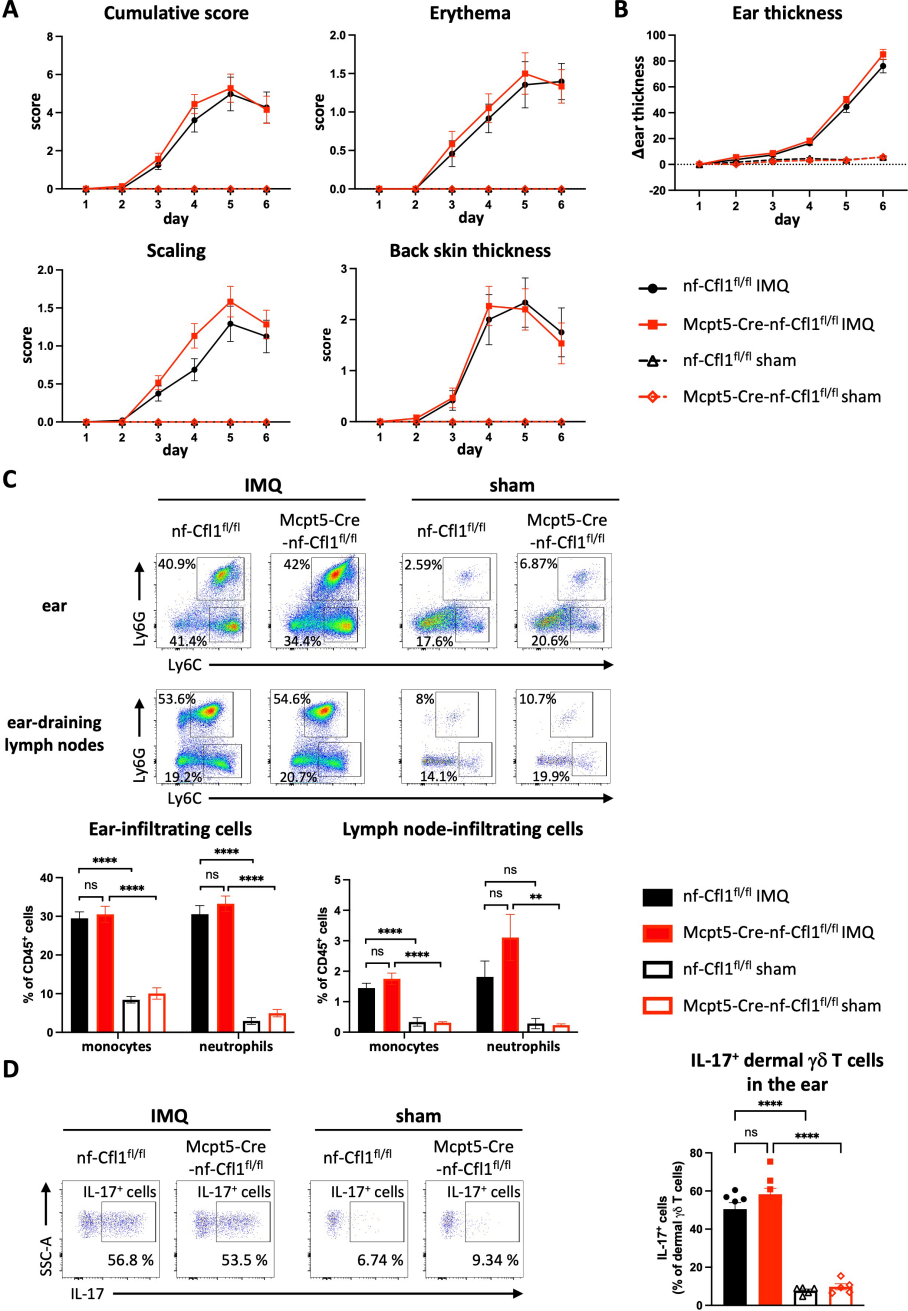
CTMC deficiency does not significantly affect the development of imiquimod-induced psoriasis in Mcpt5-Cre-nf-Cfl1^fl/fl^ mice. Mcpt5-Cre-nf-Cfl1^fl/fl^ knock-in mice and nf-Cfl1^fl/fl^ littermate controls were treated with Aldara cream (containing 5% imiquimod (IMQ)) or sham cream for 5 consecutive days. **(A)** Single scores (erythema, scaling, and thickness of back skin) and cumulative scores of IMQ- and sham-treated nf-Cfl1^fl/fl^ control mice (black) and Mcpt5-Cre-nf-Cfl1^fl/fl^ knock-in mice (red). Data in the graphs summarize results from 2 independent experiments with a total of ≥ 12 mice per group (IMQ-treated) or 10 mice per group (sham-treated). **(B)** Ear skin thickness of IMQ- and sham-treated nf-Cfl1^fl/fl^ control mice (black) and Mcpt5-Cre-nf-Cfl1^fl/fl^ knock-in mice (red). Graph depicts the percentage change relative to day 0. Data in the graph summarize results from 2 independent experiments with a total of ≥ 14 mice per group (IMQ-treated) or 10 mice per group (sham-treated). **(C)** Myeloid cells in ears and ear-draining lymph nodes of IMQ- and sham-treated nf-Cfl1^fl/fl^ control mice (black) and Mcpt5-Cre-nf-Cfl1^fl/fl^ knock-in mice (red) were analyzed by flow cytometry on day 6. Bar graph quantifies the percentage of living CD45^+^ cells. Monocytes were identified as single living CD45^+^ Ly6C^+^ Ly6G^-^ cells and neutrophils were identified as single living CD45^+^ Ly6C^int^ Ly6G^+^ cells. Data in the graphs summarize results from 2 independent experiments with a total of ≥ 14 mice per group (IMQ-treated) or 10 mice per group (sham-treated). **(D)** Percentage of IL-17^+^ γδ T cells (gated as single living CD45^+^ TCRγδ^+^ IL-17A^+^) in ear skin of IMQ- and sham-treated nf-Cfl1^fl/fl^ control mice (black) and Mcpt5-Cre-nf-Cfl1^fl/fl^ knock-in mice (red) was analyzed on day 6 by flow cytometry. Data in the graph summarize results from 1 experiment with a total of ≥ 7 mice per group (IMQ-treated) or 5 mice per group (sham-treated). Data in the graphs are represented as mean ± SEM. Kruskal-Wallis test with a Dunn’s post hoc test (**A** + **B**) or one-way analysis of variance with a Bonferroni post hoc test (**C** + **D**) were used to determine significant differences between groups. Differences of p ≤ 0.05 were considered to be statistically significant (**p ≤ 0.01; ***p ≤ 0.001; ****p < 0.0001). In line graphs (**A** + **B**), the statistical evaluation of differences between IMQ-treated and sham-treated groups is not shown.

To further elucidate the role of mast cells in the pathogenesis of psoriasis, we subjected CTMC-deficient Mcpt5-Cre-nf-Cfl1^fl/fl^ mice and nf-Cfl1^fl/fl^ control mice to IMQ-induced psoriasis-like dermatitis. Erythema, scaling, and thickness of the back skin were evaluated daily for a period of 6 days and were combined in a cumulative score. No statistically significant differences were observed in the individual disease parameters, and the cumulative psoriatic score was comparable in IMQ-treated Mcpt5-Cre-nf-Cfl1^fl/fl^ mice and nf-Cfl1^fl/fl^ littermate controls (**Figure 6A**; filled symbols). However, a slight, albeit not statistically significant, increase in scaling was observed in IMQ-treated Mcpt5-Cre-nf-Cfl1^fl/fl^ mice. As expected, mice treated with control (sham) cream did not develop psoriatic symptoms (**Figure 6A**; clear symbols). The changes in ear thickness were consistent with those observed in the thickness of the back skin. Additionally, no statistically significant differences were observed between IMQ-treated nf-Cfl1^fl/fl^ and Mcpt5-Cre-nf-Cfl1^fl/fl^ mice (**Figure 6B**, filled symbols). Sham-treated animals, as expected, did not show a reaction, and their ear thickness remained constant (**Figure 6B**, clear symbols).

To check if the absence of mast cells affected immune cell infiltration, immune cell populations in ears and ear-draining lymph nodes at day 6 were analyzed by flow cytometry. In parallel with the progression of the disease, there was a notable increase in the proportion of monocytes and neutrophils in IMQ-treated animals in comparison to sham-treated mice, both in ear tissue and ear-draining lymph nodes (**Figure 6C**). A comparison between IMQ-treated CTMC-deficient Mcpt5-Cre-nf-Cfl1^fl/fl^ mice and IMQ-treated nf-Cfl1^fl/fl^ control mice revealed no significant differences in the frequency of monocytes and neutrophils in ear tissue or ear-draining lymph nodes (**Figure 6C**, filled bars). By trend, higher neutrophil frequencies were observed in the ear-draining lymph nodes of IMQ-treated Mcpt5-Cre-nf-Cfl1^fl/fl^ mice. However, these differences did not reach statistical significance.

As previously mentioned, IL-17 is a key mediator in the pathogenesis of psoriasis (reviewed in (24)). Th17 cells and IL-17-producing γδ T cells in the skin are critical disease drivers in IMQ-induced psoriasis. Consequently, an analysis of IL-17-producing cells in the ear tissue was conducted to check if mast cell deficiency might affect them. The percentage of IL-17-producing γδ T cells was strongly increased in IMQ-treated groups compared to sham-treated groups (**Figure 6D**). However, only a slight but not significant increase in IL-17-producing γδ T cells was observed in IMQ-treated Mcpt5-Cre-nf-Cfl1^fl/fl^ mice as compared to nf-Cfl1^fl/fl^ mice (**Figure 6D**, filled bars).

In conclusion, mast cell-deficient Mcpt5-Cre-nf-Cfl1^fl/fl^ knock-in mice did not show a significantly altered susceptibility to IMQ-induced psoriasis compared to nf-Cfl1^fl/fl^ control mice.

### 3.8 Clearing of vaccinia virus skin infection is not dependent on CTMCs

**Figure 7:**
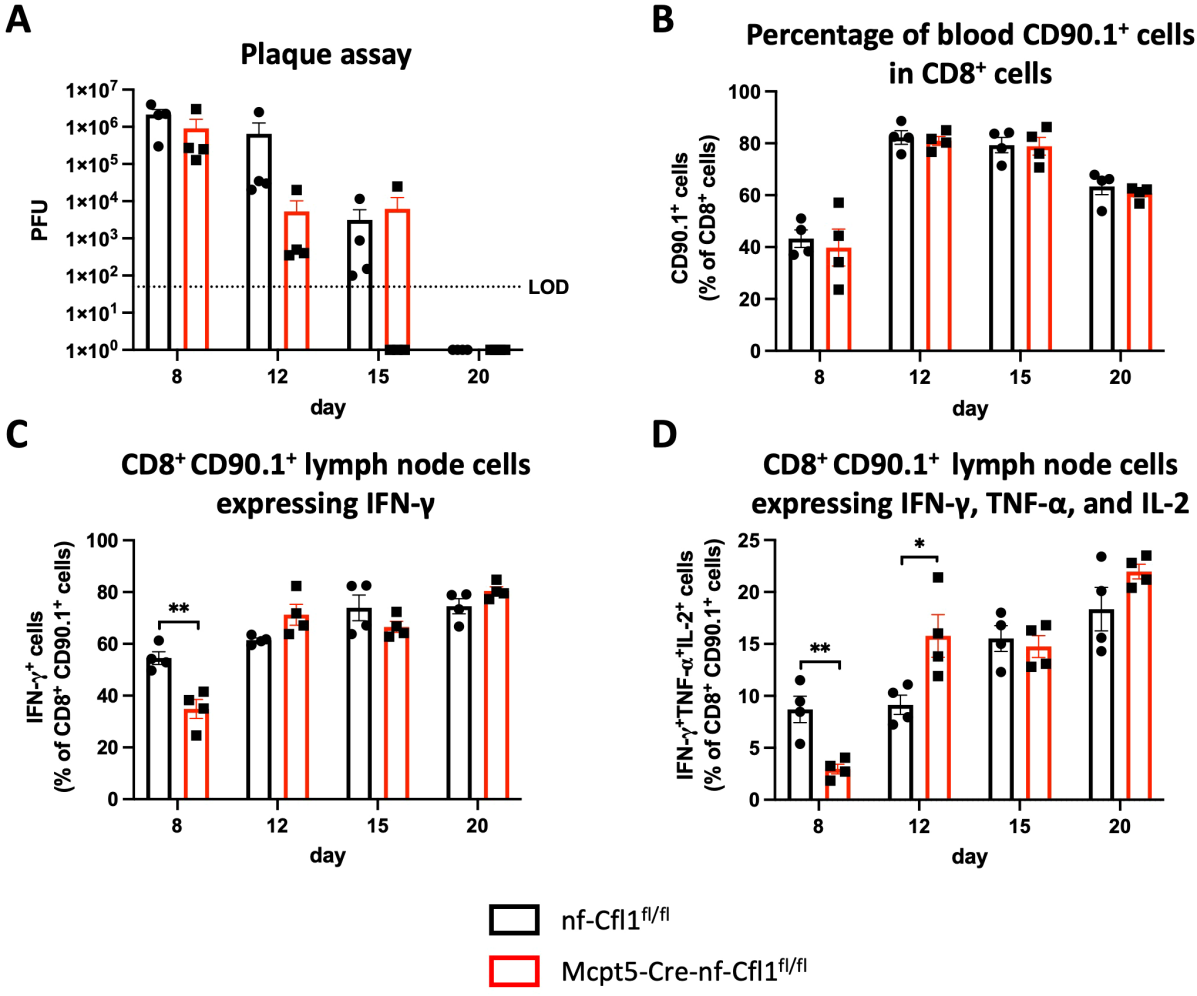
Clearance of vaccinia virus infection in Mcpt5-Cre-nf-Cfl1^fl/fl^ knock-in mice is comparable to nf-Cfl1^fl/fl^ control mice. 1 x 10^5^ GP_33–41_-specific Thy1.1+/+ (CD90.1^+^) T cells were transferred intravenously into nf-Cfl1^fl/fl^ control mice and Mcpt5-Cre-nf-Cfl1^fl/fl^ knock-in mice. On the subsequent day, mice were locally infected at the dorsal part of both ears with 2 × 10^6^ plaque-forming units recombinant vaccinia virus expressing GP_33–41_ per ear. Afterwards, the ear skin area was punctured 25 times with a 28G × ½” needle. Values were obtained on days 8, 12, 15, and 20 post-infection. **(A)** The virus titer in ears of nf-Cfl1^fl/fl^ control mice (black) and Mcpt5-Cre-nf-Cfl1^fl/fl^ knock-in mice (red) was determined by plaque assay over time. **(B)** The percentage of Thy1.1+ (CD90.1^+^) cells among CD8^+^ cells in the blood of nf-Cfl1^fl/fl^ control mice (black) and Mcpt5-Cre-nf-Cfl1^fl/fl^ knock-in mice (red) was determined by flow cytometry. **(C, D)** Ear-draining lymph node cells from nf-Cfl1^fl/fl^ control mice (black) and Mcpt5-Cre-nf-Cfl1^fl/fl^ knock-in mice (red) infected with vaccinia virus were restimulated *ex vivo* for 6 h with GP_33–41_ and treated with Brefeldin A to inhibit cytokine secretion. The frequency of IFN-γ^+^**(C)** and IFN-γ^+^ TNF-α^+^ IL-2^+^ cells **(D)** in CD90.1^+^ CD8^+^ cells was analyzed by flow cytometry. Data in the bar graphs are represented as mean ± SEM and summarize results from 1 experiment with a total of 4 mice per group per time point. Unpaired Student’s t-test was used to determine significant differences between groups. Differences of p ≤ 0.05 were considered to be statistically significant (*p ≤ 0.05; **p ≤ 0.01).

Vaccinia virus, a member of the poxvirus family, can infect the skin and can cause lesions or rashes. It has been utilized as a live-virus vaccine against smallpox infection, contributing to the eradication of the disease in the late 1970s (25). Wang et al. demonstrated that mast cell-deficient Kit^wsh-/-^ mice exhibited increased susceptibility to skin infection with vaccinia virus in comparison to wildtype animals (26). Given the plethora of abnormalities observed in c-KIT-dependent mast cell-deficient mouse models irrespective of the mast cell compartment (reviewed in (27)), we employed our mast cell-deficient Mcpt5-Cre-nf-Cfl1^fl/fl^ mice to investigate the role of mast cells in the clearance of vaccinia virus infection. To be able to monitor antigen-specific CD8^+^ T cell responses, we used a recombinant vaccinia virus expressing the lymphocytic choriomeningitis virus glycoprotein-derived epitope GP_33–41_ (rVV-GP33) and adoptively transferred CD90.1^+^ CD8^+^ TCR327 transgenic T cells into Mcpt5-Cre-nf-Cfl1^fl/fl^ and nf-Cfl1^fl/fl^ mice. These CD90.1^+^ CD8^+^ T cells were isolated from TCR327 mice and carry a transgenic T cell receptor recognizing GP_33–41_ presented by H2-D^b^. To establish infection, rVV-GP33 was applied topically to the ear skin of Mcpt5-Cre-nf-Cfl1^fl/fl^ and nf-Cfl1^fl/fl^ mice. At days 8, 12, 15, and 20 post-infection, virus titers in the ear were determined via plaque assay. Additionally, immune cells were isolated from the ear skin, ear-draining lymph nodes, blood, and spleen for flow cytometric analysis. The virus titer was comparable between Mcpt5-Cre-nf-Cfl1^fl/fl^ and nf-Cfl1^fl/fl^ mice at all analyzed time points (**Figure 7A**). The highest virus titer was detected at the earliest measurement time point, 8 days post-infection, and subsequently declined over time, until no virus was detectable at 20 days post-infection. Please note that data for Mcpt5-Cre-nf-Cfl1^fl/fl^ mice obtained for day 15 showed considerable biological variability.

The frequency of antigen-specific CD90.1^+^ CD8^+^ T cells in the blood of Mcpt5-Cre-nf-Cfl1^fl/fl^ and nf-Cfl1^fl/fl^ mice was comparable at all analyzed time points (**Figure 7B**). At day 8 post-infection, approximately 40% of all CD8^+^ blood T cells were specific for the recombinant vaccinia virus, as represented by CD90.1^+^ cells. This value increased to over 80% at day 12 post-infection and subsequently declined at a gradual rate.

To determine whether CTMCs play a functional role in the cytotoxic T lymphocyte response against vaccinia virus-mediated skin infection, cells from skin-draining lymph nodes were restimulated *in vitro* with the recombinant virus-specific antigen GP_33–41_ and the production of the cytokines IFN-γ, TNF-α, and IL-2 was analyzed. At day 8 post-infection, a significantly higher number of antigen-specific CD8^+^ cells from nf-Cfl1^fl/fl^ mice than from Mcpt5-Cre-nf-Cfl1^fl/fl^ mice produced IFN-γ (**Figure 7C**). At days 12, 15, and 20 post-infection, no significant differences in the frequency of IFN-γ-producing antigen-specific CD8^+^ cells in nf-Cfl1^fl/fl^ versus Mcpt5-Cre-nf-Cfl1^fl/fl^ mice were observed. The analysis of antigen-specific CD8^+^ cells producing IFN-γ plus TNF-α and IL-2 yielded comparable results. At day 8, a significantly greater number of antigen-specific CD8^+^ cells from nf-Cfl1^fl/fl^ produced IFN-γ, TNF-α, and IL-2 than those from Mcpt5-Cre-nf-Cfl1^fl/fl^ mice (**Figure 7D**). However, at day 12 post-infection, the frequency of IFN-γ^+^ TNF-α^+^ IL-2^+^ antigen-specific CD8^+^ cells was slightly higher in Mcpt5-Cre-nf-Cfl1^fl/fl^ than in nf-Cfl1^fl/fl^ mice. In conclusion, a delayed IFN-γ response of antigen-specific CD8^+^ T cells in the draining lymph nodes was observed at day 8 after infection of mast cell-deficient Mcpt5-Cre-nf-Cfl1^fl/fl^ mice. However, the clearance of vaccinia virus-induced skin infection appeared unaffected in the absence of CTMCs.

## 4 Discussion

The aim of this study was to determine the role of the actin remodeling protein Cfl1 for CTMCs in mice. For this purpose, floxed nf-Cfl1^fl/fl^ mice were cross-bred with Mcpt5-Cre mice resulting in the generation of Mcpt5-Cre-nf-Cfl1^fl/fl^ mice which are expressing nf-Cfl1 instead of the wildtype protein specifically in CTMCs.

The expression of nf-Cfl1 instead of wildtype Cfl1 under the control of the CTMC-specific Mcpt5 promoter resulted in the generation of a mouse devoid of CTMCs, as evidenced by the absence of these cells in the peritoneal cavity, back skin, and ear skin. These Mcpt5-Cre-nf-Cfl1^fl/fl^ mice did not develop a systemic anaphylactic reaction, confirming the absence of CTMCs also functionally.

### 4.1 Mast cell-deficient mouse models

The analysis of immune cell compartments in the spleen revealed comparable numbers of B cells, T cells, DCs, neutrophils, eosinophils, macrophages, and basophils in CTMC-deficient Mcpt5-Cre-nf-Cfl1^fl/fl^ mice and nf-Cfl1^fl/fl^ control mice. NK cells were the only analyzed cell type being significantly diminished in the spleens of Mcpt5-Cre-nf-Cfl1^fl/fl^ mice, exhibiting a reduction by around 35% relative to nf-Cfl1^fl/fl^ mice. However, no differences were observed in the lymph nodes of the same mice. Dudeck et al. demonstrated that Mcpt5-Cre-mediated recombination of floxed alleles occurred in more than 10% of peripheral blood NK cells (11). We observed a distinct eGFP^+^ population in splenic NK cells isolated from heterozygous Mcpt5-Cre-nf-Cfl1^wt/fl^ mice (**Supplementary Figure 3**). Consequently, ectopic expression of nf-Cfl1 in NK cells is likely the underlying cause for the diminished splenic NK cell number observed in Mcpt5-Cre-nf-Cfl1^fl/fl^ mice. This finding suggests that functional Cfl1 is critical for splenic NK cells in mice. This should be considered when employing Mcpt5-Cre-nf-Cfl1^fl/fl^ mice in NK cell-related disease models.

The lack of CTMCs in Mcpt5-Cre-nf-Cfl1^fl/fl^ mice most likely results from a defect in CTMC development, not from death of mature mast cells. This conclusion is based on experiments performed with ROSA26-CreERT2-nf-Cfl1^fl/fl^ mice that allow the temporal expression of nf-Cfl1 in mature cells *in vitro* through the administration of tamoxifen, with concomitant knock-out of wildtype Cfl1. Thus, the induction of homozygous nf-Cfl1 expression in fully developed mast cells isolated from the peritoneum of these mice did not result in increased cell death. Note that unlike in mast cells, homozygous expression of nf-Cfl1 in splenic T cells resulted in death of nearly all T cells. These results show that functional Cfl1 is essential for the survival of mature T cells but not for the survival of fully developed mast cells. Collectively, our data imply that mechanisms other than apoptosis account for the reduced CTMC numbers in Mcpt5-Cre-nf-Cfl1^fl/fl^ mice. Cfl1, as an actin-severing protein, is a plausible regulator of multiple processes critical for mast cell biology, including differentiation, proliferation, and tissue homing. Preliminary data obtained with bone marrow-derived mast cells from Mcpt5-Cre-nf-Cfl1^fl/fl^ mice indicated reduced outgrowth compared with bone marrow-derived mast cells from control mice. This is consistent with a developmental impairment. However, we cannot exclude the possibility that impaired mobilization of mast cell progenitors from bone marrow to connective tissues also contributes. Future experiments with extended *in vitro* differentiation assays, progenitor tracking, and homing studies will be required to define the precise step(s) affected by Cfl1 deficiency that cause the absence of CTMCs in Mcpt5-Cre-nf-Cfl1^fl/fl^ mice.

One limitation of our study is the absence of functional data addressing the function of nf-Cfl1 in MMCs. MMCs are present in only very low numbers under steady-state conditions, rendering their direct analysis technically challenging. Experimental expansion of MMCs, for example in the small intestine through parasite infection, as described by Rodewald and colleagues (12), would enable clearer visualization. Nevertheless, the Mcpt5-Cre system used here has already been thoroughly validated to specifically deplete CTMCs while sparing MMCs (10, 12, 28). This supports our conclusion that the observed phenotypes reflect CTMC-specific functions.

The complete lack of CTMCs combined with normal numbers of basophils is a specific feature of the Mcpt5-Cre-nf-Cfl1^fl/fl^ mice investigated here. As outlined in **Table 1**, a variety of mouse lines exhibiting mast cell deficiency have been described. c-KIT mutant mice represented the first generation of mast cell-deficient mice. These mice carry mutations in the gene encoding the receptor tyrosine kinase c-KIT, which is a surface marker typically expressed on mast cells, as well as on hematopoietic stem and progenitor cells. Rodewald and Feyerabend provided a comprehensive overview of the limitations of c-KIT-dependent mast cell-deficient mouse models, emphasizing a multitude of abnormalities that extend beyond the mast cell compartment (27).

**Table 1:** Overview of mast cell-deficient mouse models.

Mouse line	Genotype	Mast cell presence	Limitations	Relevant publication(s)
Kit^W-sh/W-sh^ mice	c-KIT deficiency	profound deficiency in mast cells	neutrophilia, megakaryocytosis, thrombocytosis, splenomegaly, cardiac hypertrophy	(29) (30)
Kit^W/Wv^ mice	c-KIT deficiency	markedly reduced levels of tissue mast cells	sterility, multiple hematopoietic abnormalities (e.g., impaired T cell development in the thymus)	(29)
Cre-Master Cpa3^Cre/+^ mice	Cre expression in Cpa3^+^ cells ➔ “Cre toxicity”	no mucosal and connective tissue mast cells	60% reduction in splenic basophils	(12)
HELLO KITTY Cpa3-Cre; Mcl-1^fl/fl^ mice	knock-out of anti-apoptotic factor Mcl-1 in Cpa3^+^ cells	no mucosal and connective tissue mast cells	60–75% reduction in basophils	(31)
Mcpt5^Cre^ iDTR mice	diphtheria toxin receptor expression on Mcpt5^+^ cells ➔ injection of diphtheria toxin leads to ablation of mast cells	inducible, local ablation of connective tissue mast cells	several rounds of diphtheria toxin application necessary for depletion of skin mast cells	(11)
Mcpt5^Cre^ R-DTA mice	diphtheria toxin expression in Mcpt5^+^ cells	significantly reduced connective tissue mast cell numbers	about 10% of mast cells still present in abdominal and back skin	(11)
Mas-TRECK mice	diphtheria toxin receptor expression under control of an intronic enhancer that regulates IL-4 expression in mast cells and basophils	inducible ablation of mucosal and connective tissue mast cells	several rounds of diphtheria toxin application necessary for depletion of mast cells; complete depletion of basophils	(32)
Mcpt5/Cma1^DTR^ mice	diphtheria toxin receptor expression in Mcpt5^+^ cells	inducible ablation of connective tissue mast cells	several rounds of diphtheria toxin application necessary for depletion of connective tissue mast cells; 25% of marginal zone B cells are killed by diphtheria toxin treatment	(33)
red mast cells and basophil (RMB) mice	diphtheria toxin receptor expression under control of the endogenous promoter of Ms4a2, encoding FcεRI β chain in mast cells and basophils	inducible ablation of mast cells and basophils	several rounds of diphtheria toxin application necessary for depletion of mast cells and basophils; necessary to wait for 12 days after last diphtheria toxin administration for repopulation of basophils	(34)
Mcpt5Cre/Dicer^fl/fl^ mice	knock-out of endoribonuclease Dicer in Mcpt5^+^ cells	significantly reduced connective tissue mast cell numbers	about 15% of mast cells still present in peritoneal cavity	(35)
GATA2^f/f^ Mcpt5-Cre mice	knock-out of transcription factor GATA2 in Mcpt5^+^ cells	nearly complete deficiency of mast cells in ear skin, peritoneum, stomach, and trachea		(36)
Chm-Cre; Mcl-1^fl/fl^ mice	knock-out of anti-apoptotic factor Mcl-1 in Chm^+^ cells	markedly reduced numbers of mucosal mast cells in mucosal tissues		(37)
Mcpt5-Cre-nf-Cfl1^fl/fl^ mice	expression of non-functional cofilin-1 in Mcpt5^+^ cells	no connective tissue mast cells	modest reduction of NK cells in the spleen	present manuscript

In 2011, Feyerabend, Lilla, and Dudeck published the first studies about various c-KIT-independent mouse models for mast cell deficiency. In Cre-Master Cpa3^Cre/+^ mice, Cre expression under the control of the carboxypeptidase A3 (Cpa3) promoter resulted in Cre toxicity and the absence of MMCs and CTMCs (12). In these mice, the number of splenic basophils was also reduced by 60%. Also HELLO KITTY (Cpa3-Cre; Mcl-1^fl/fl^) mice had markedly reduced numbers of both mast cells and basophils (31). Using Mcpt5-Cre mice, an inducible and a constitutive mouse model for CTMC deficiency were generated (11). The Mcpt5^Cre^ iDTR model is an inducible mouse model based on CTMC-specific expression of the diphtheria toxin receptor. Mast cell depletion necessitates the administration of multiple rounds of diphtheria toxin. These diphtheria toxin treatments are not necessary in the constitutive Mcpt5^Cre^ R-DTA mouse model, in which the constitutive expression of diphtheria toxin in CTMCs results in a markedly reduced mast cell number. However, in these mice, 10% of the normal mast cell numbers were still present in the abdominal and back skin. Mas-TRECK mice express the diphtheria toxin receptor under control of an intronic enhancer that regulates IL-4 expression in mast cells and basophils (32). In these mice, administration of diphtheria toxin leads to the ablation of mast cells and basophils. Mcpt5/Cma1^DTR^ mice are another mouse model for inducible CTMC deficiency (33). Expression of the diphtheria toxin receptor under control of the CTMC-specific Mcpt5 promoter allows the depletion of CTMCs after several rounds of diphtheria toxin administration. However, 25% of marginal zone B cells are killed by this diphtheria toxin treatment. In red mast cells and basophil (RMB) mice, the diphtheria toxin receptor is expressed under the control of the endogenous promoter of Ms4a2, which is encoding the FcεRI β chain in mast cells and basophils (34). Diphtheria toxin treatment leads to the depletion of mast cells and basophils. After a period of 12 days, basophils were repopulated and the mice could be used as mast cell-deficient mice. In Mcpt5-Cre/Dicer^fl/fl^ mice, the endoribonuclease Dicer is knocked-out in Mcpt5-expressing cells, resulting in a mouse with markedly reduced numbers of CTMCs (35). In GATA2^f/f^ Mcpt5-Cre mice, the transcription factor GATA2 is knocked-out in Mcpt5-expressing cells, leading to a nearly complete deficiency of mast cells in ear skin, peritoneum, stomach, and trachea (36). In Chm-Cre; Mcl-1^fl/fl^ mice, the anti-apoptotic factor Mcl1 is knocked-out under the control of the baboon α-chymase promoter which is specifically active in MMCs. These mice have markedly reduced numbers of MMCs (37).

### 4.2 The role of mast cells in inflammatory skin diseases

The role of mast cells in inflammatory skin diseases is still under debate. In this context, molecules such as major histocompatibility complex class II proteins are known to be transferred from DCs to mast cells, thereby enhancing the T cell priming capacity of mast cells (38). Furthermore, Dudeck et al. have demonstrated a promoting role for mast cells during DNFB- and fluorescein isothiocyanate-induced CHS (11). They observed a significantly reduced ear thickness and a diminished infiltration of CD45^+^ cells and T cells into the ear skin 24 h after challenge. Otsuka et al. demonstrated that the ear swelling reaction in DNFB- and oxazolone-induced CHS in inducible mast cell-deficient Mas-TRECK mice was also significantly reduced compared to control mice 24 h and 48 h after challenge (39). In contrast, in the present study, the ear thickness of CTMC-deficient Mcpt5-Cre-nf-Cfl1^fl/fl^ mice 8 h and 24 h after DNFB challenge did not differ significantly from that of control animals. Furthermore, no alteration in the infiltration of immune cells into the ear skin was observed. These discrepancies may be attributed to the utilization of varying concentrations of DNFB and protocols to elicit CHS. Dudeck et al. employed 0.5% DNFB for sensitization and 0.2% DNFB for challenge, which was performed six days after sensitization. Otsuka et al. sensitized their mice with 0.5% DNFB and challenged them five days later with 0.3% DNFB. In contrast, we sensitized and challenged the mice with 0.25% DNFB, respectively, with the challenge occurring five days after sensitization. In line with these discrepancies, it has been demonstrated that mast cells can function either as pro-inflammatory or anti-inflammatory cells during CHS reactions, depending on the severity of the applied model (28). This results in either an exacerbated or diminished degree of ear swelling in mast cell-deficient mice. Such a potential divergent role of mast cells in CHS reactions has been reviewed by Gaudenzio et al., who state that mast cells may strengthen CHS in mild models and restrain it in more severe models (40). The findings of our study can therefore only be interpreted within the specific CHS conditions applied here.

The existing literature contains conflicting data regarding the involvement of mast cells in the IMQ-induced psoriasis-like dermatitis model. One study reported unchanged mast cell numbers in the skin of IMQ-treated wildtype mice (41), while another demonstrated an accumulation of dermal mast cells upon IMQ treatment (42). In mast cell-deficient Kit^W-sh/W-sh^ mice, daily treatment with the Toll-like receptor 7 agonist IMQ resulted in a decreased ear swelling reaction during the first three days compared to wildtype littermates (43). This effect was gone if Kit^W-sh/W-sh^ mice were reconstituted with bone marrow-derived mast cells, leading to the hypothesis that mast cells are required for the fast inflammatory response to Toll-like receptor 7 ligation. However, as discussed by Rodewald and Feyerabend, c-KIT-dependent mast cell-deficient mice reconstituted with bone marrow-derived mast cells can behave differently from mast cell-deficient mice which are wildtype for c-KIT (27). Our study, in which c-KIT-independent CTMC-deficient Mcpt5-Cre-nf-Cfl1^fl/fl^ mice were employed, shows that the absence of mast cells did not influence the induction of psoriatic symptoms following treatment of mice with IMQ, as indicated by the cumulative clinical score, which comprises the scores for back skin erythema, scaling, and thickness. Dermal IL-17-producing γδ T cells did not differ between groups. These cells are considered to play an important role in the pathogenesis of psoriasis in the IMQ-induced mouse model, since γδ T cell-deficient mice show significantly decreased symptoms, whereas αβ T cell-deficient mice show unaffected reactions (44, 45). Taken together, these results imply that skin mast cells are not required for the development of psoriatic symptoms in the IMQ-induced psoriasis-like model.

A different situation appears to exist in human skin. In psoriasis patients, more IL-17-producing mast cells and neutrophils than IL-17-producing T cells were found in psoriatic lesions (23). Additional key cytokines in psoriasis are IL-23 and IL-22 (46, 47). Psoriasis patients exhibited increased levels of IL-17 and IL-22 levels in both the skin and the blood. Mashiko et al. showed that in humans, mast cells are the primary producers of IL-22 in psoriatic plaques, which is why they suggested a promoting role for mast cells in psoriasis (48). This assumption was underlined by a recent publication demonstrating that a significant proportion of mast cells is activated in psoriatic human skin, as revealed by high-throughput transcriptomic data analysis (49). Consequently, the authors concluded a progressive role of mast cells in the onset of psoriasis in patients. Thus, differences between mouse models of psoriasis and human psoriasis need to be further addressed.

The involvement of mast cells in the clearance of viral infections remains a subject of debate. For instance, mast cell-deficient Kit^W-sh/W-sh^ mice exhibited an increased susceptibility to skin infection with vaccinia virus relative to wildtype mice (26). The size of the skin lesions and the virus titer were significantly increased in Kit^W-sh/W-sh^ mice compared to wildtype mice. This finding led to the conclusion that mast cells may play a protective role in viral skin infections by releasing antimicrobial peptides. However, in our experimental setting with c-KIT-independent CTMC-deficient Mcpt5-Cre-nf-Cfl1^fl/fl^ mice, no significant differences in vaccinia virus titers were observed at days 8, 12, 15, and 20 post-infection, as compared to control mice. Although it cannot be completely excluded that monitoring the virus titer as early as 1 or 2 days after infection might reveal potential differences in the onset of vaccinia virus infection in mast cell-deficient mice compared to control mice, mast cells do not appear to be critically involved in the clearance of vaccinia virus infections. In line with this assumption, other studies using c-KIT-independent mouse models have demonstrated that mast cells do not appear to play a key role in the clearance of viral infections. For instance, human papilloma virus-induced epithelial hyperplasia and angiogenesis remained unaltered in Mcpt5^Cre^ R-DTA mice lacking mast cells (50).

In summary, the present study demonstrates that the targeted expression of a non-functional form of Cfl1 in CTMCs, with concomitant knock-out of wildtype Cfl1, leads to the complete absence of CTMCs in Mcpt5-Cre-nf-Cfl1^fl/fl^ mice, highlighting an essential role of Cfl1 in CTMC development. Besides a modest decrease in splenic NK cells, likely attributable to ectopic expression of nf-Cfl1, all other immune cells, including basophils, were present in normal numbers. Functionally, Mcpt5-Cre-nf-Cfl1^fl/fl^ mice exhibited impaired systemic anaphylaxis responses, confirming a critical role for CTMCs in this context. However, these mice responded normally in models of CHS, IMQ-induced psoriasis-like dermatitis, and clearance of vaccinia virus-induced skin infection, indicating that CTMCs are dispensable in these inflammatory skin conditions. The maintenance of basophil populations in the absence of CTMCs in Mcpt5-Cre-nf-Cfl1^fl/fl^ mice may contribute to the observed differences in disease outcomes compared to other mast cell-deficient models in which basophils are often reduced or absent. The precise role of basophils in the development of the aforementioned diseases needs to be further investigated.

Consequently, Mcpt5-Cre-nf-Cfl1^fl/fl^ mice, which selectively lack CTMCs, serve as an excellent mouse model to study the specific role of mast cells in disease. In addition to chronic inflammatory diseases, mast cells are now recognized as active participants in neuroimmune crosstalk, neurogenic inflammation, itch, and pain—all areas of growing research interest (see (51)). Our model thus provides a precise tool to study the interaction of mast cells with neuronal pathways and other immune cells in health and disease.

## Data Availability

The raw data supporting the conclusions of this article will be made available by the authors, without undue reservation.
