# Supplementary material for: Inactivation of cofilin-1 in Mcpt5-Cre-nf-Cfl1fl/fl mice prevents the formation of connective tissue mast cells without affecting basophils: a new tool to investigate the specific role of CTMCs in disease
Source: Front Immunol. 2026 Jan 28;16:1671735. doi: 10.3389/fimmu.2025.1671735 (PMC12891179; doi:10.3389/fimmu.2025.1671735)
Supplement: Supplementary file 1 [file Presentation1.pptx]

## Slide 1
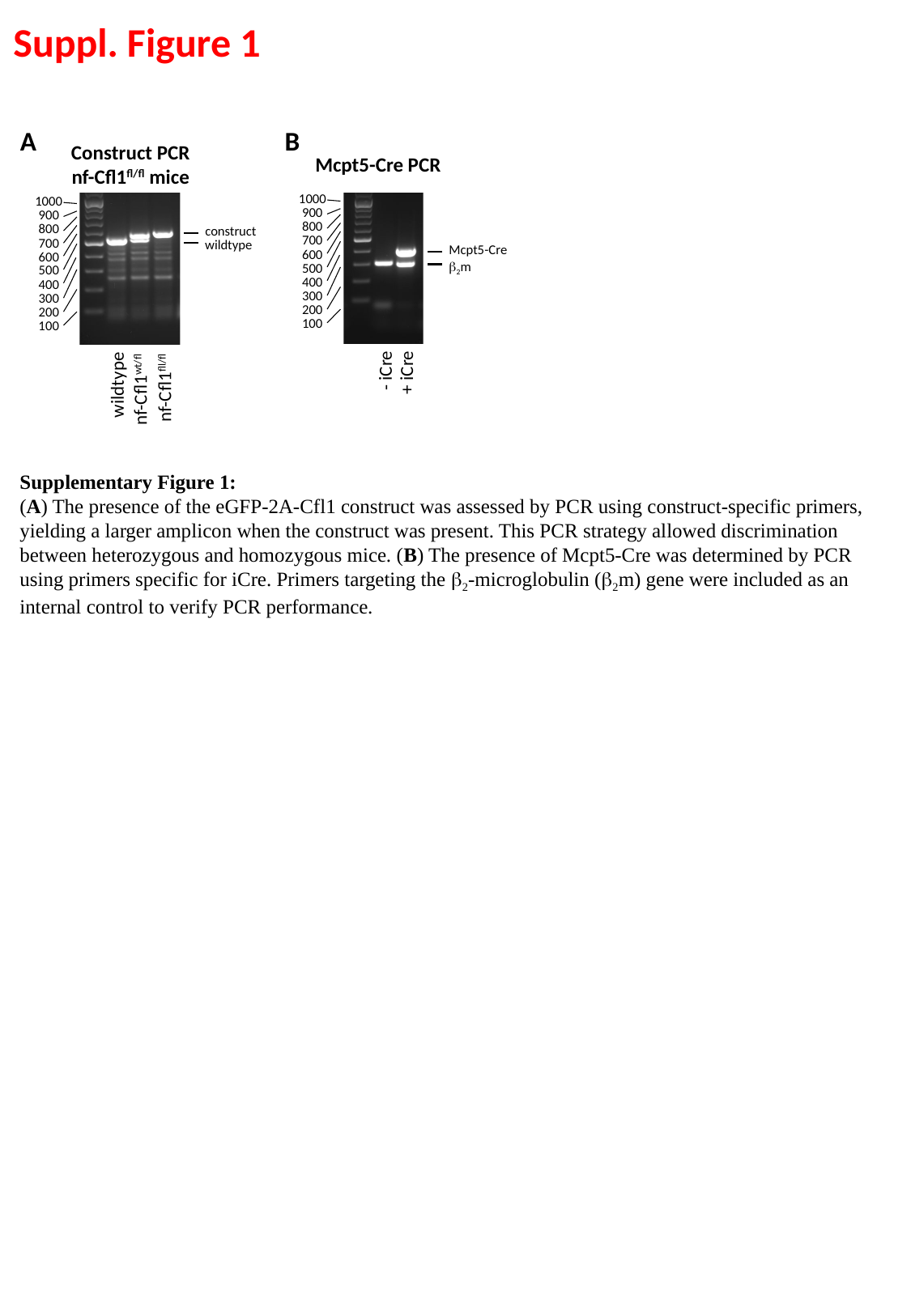

Suppl. Figure 1
A
B
Construct PCR
nf-Cfl1fl/fl mice
Mcpt5-Cre PCR
1000
1000
900
900
800
800
construct
700
700
wildtype
Mcpt5-Cre
600
600
β2m
500
500
400
400
300
300
200
200
100
100
- iCre
+ iCre
wildtype
nf-Cfl1fll/fl
nf-Cfl1wt/fl
Supplementary Figure 1:
(A) The presence of the eGFP-2A-Cfl1 construct was assessed by PCR using construct-specific primers, yielding a larger amplicon when the construct was present. This PCR strategy allowed discrimination between heterozygous and homozygous mice. (B) The presence of Mcpt5-Cre was determined by PCR using primers specific for iCre. Primers targeting the β2-microglobulin (β2m) gene were included as an internal control to verify PCR performance.

## Slide 2
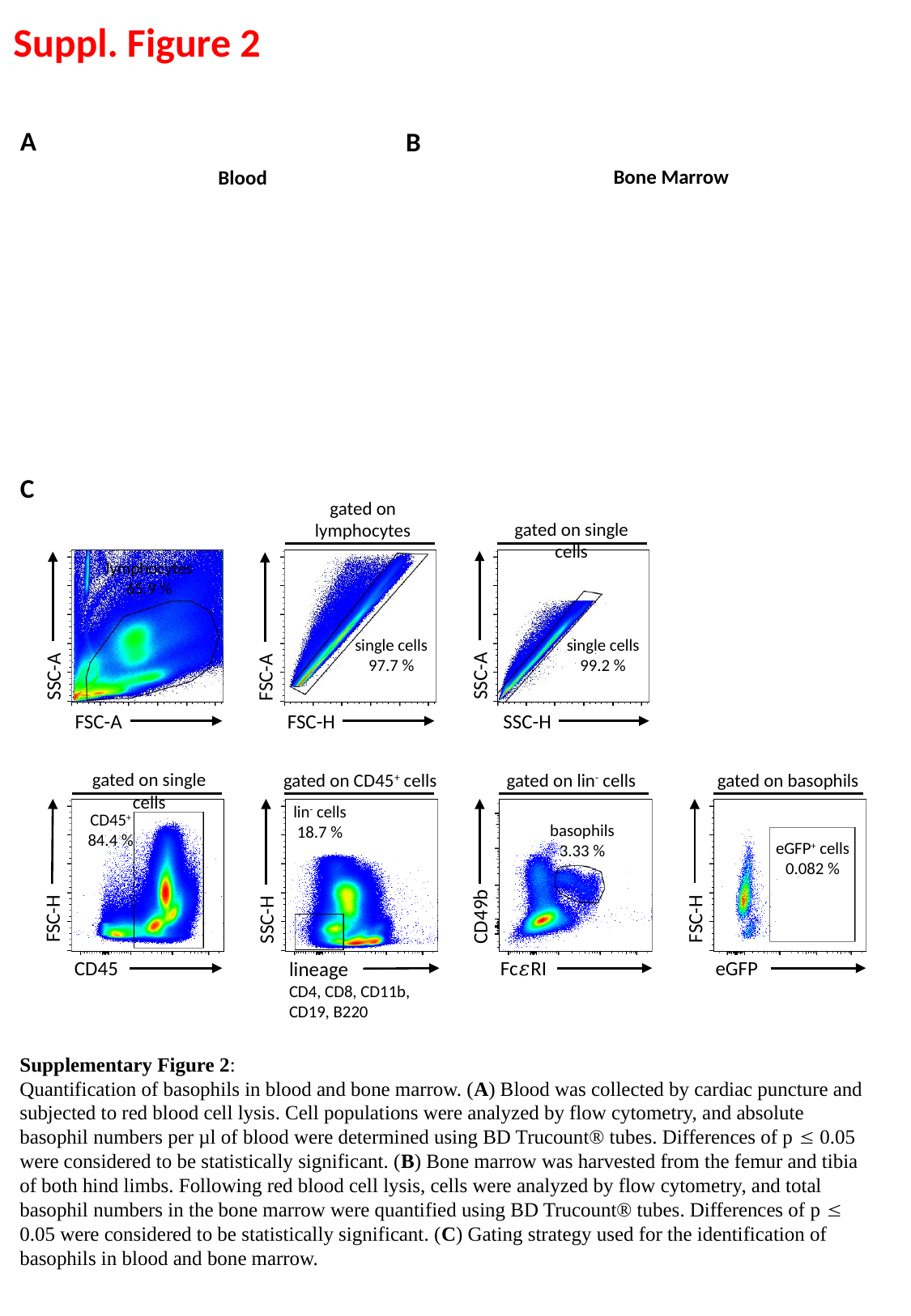

Suppl. Figure 2
A
B
Bone Marrow
Blood
C
gated on lymphocytes
gated on single cells
lymphocytes
65.9 %
single cells
97.7 %
single cells
99.2 %
SSC-A
SSC-A
FSC-A
FSC-A
FSC-H
SSC-H
gated on single cells
gated on CD45+ cells
gated on lin- cells
gated on basophils
lin- cells
18.7 %
CD45+
84.4 %
basophils
3.33 %
eGFP+ cells
0.082 %
FSC-H
FSC-H
SSC-H
CD49b
CD45
Fc𝜀RI
eGFP
lineage
CD4, CD8, CD11b,
CD19, B220
Supplementary Figure 2:
Quantification of basophils in blood and bone marrow. (A) Blood was collected by cardiac puncture and subjected to red blood cell lysis. Cell populations were analyzed by flow cytometry, and absolute basophil numbers per µl of blood were determined using BD Trucount® tubes. Differences of p ≤ 0.05 were considered to be statistically significant. (B) Bone marrow was harvested from the femur and tibia of both hind limbs. Following red blood cell lysis, cells were analyzed by flow cytometry, and total basophil numbers in the bone marrow were quantified using BD Trucount® tubes. Differences of p ≤ 0.05 were considered to be statistically significant. (C) Gating strategy used for the identification of basophils in blood and bone marrow.

## Slide 3
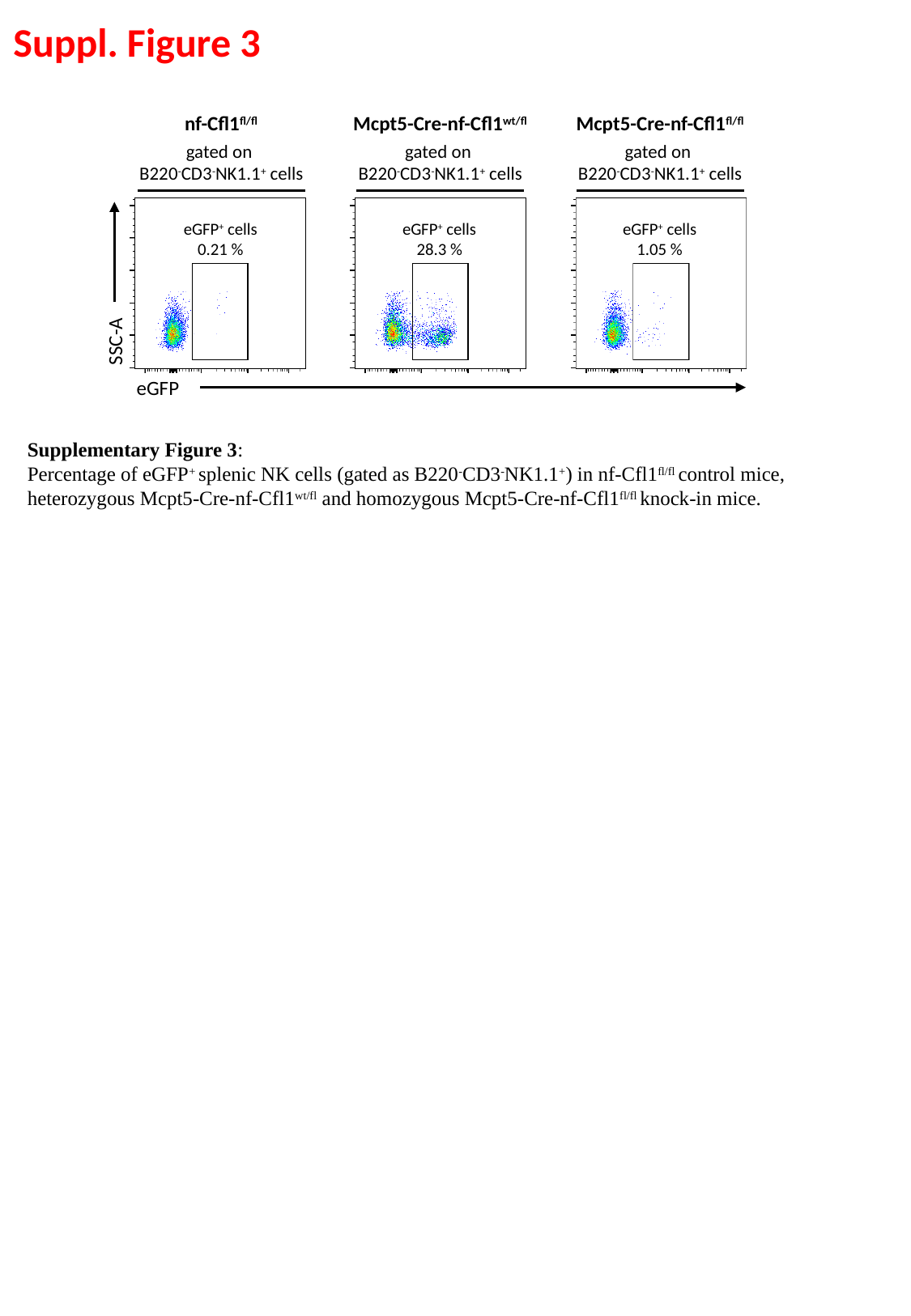

Suppl. Figure 3
nf-Cfl1fl/fl
Mcpt5-Cre-nf-Cfl1wt/fl
Mcpt5-Cre-nf-Cfl1fl/fl
gated on
B220-CD3-NK1.1+ cells
gated on
B220-CD3-NK1.1+ cells
gated on
B220-CD3-NK1.1+ cells
eGFP+ cells
0.21 %
eGFP+ cells
28.3 %
eGFP+ cells
1.05 %
SSC-A
eGFP
Supplementary Figure 3:
Percentage of eGFP+ splenic NK cells (gated as B220-CD3-NK1.1+) in nf-Cfl1fl/fl control mice, heterozygous Mcpt5-Cre-nf-Cfl1wt/fl and homozygous Mcpt5-Cre-nf-Cfl1fl/fl knock-in mice.
